# A Revised Phylogeny of the *Mentha spicata* Clade Reveals Cryptic Species

**DOI:** 10.3390/plants10040819

**Published:** 2021-04-20

**Authors:** Olivier C. G. Heylen, Nicolas Debortoli, Jonathan Marescaux, Jill K. Olofsson

**Affiliations:** 1 Hof ter Laken, Hofdreef 5, B-2221 Booischot, Belgium; 2 E-BIOM SA, 5/7 Rue Godefroid, B-5000 Namur, Belgium; n.debortoli@e-biom.com (N.D.); j.marescaux@e-biom.com (J.M.); 3 Section for GeoGenetics, GLOBE Institute, University of Copenhagen, Øster Farimagsgade 5, bygning 7, DK-1353 Copenhagen, Denmark; jill.olofsson@sund.ku.dk

**Keywords:** cryptic species, discriminant analysis, hyperdiversity, ITS sequences, *Mentha*, morphometrics, phylogenetics, porous genomes, SCoT, *Spicatae*

## Abstract

The genus *Mentha* is taxonomically and phylogenetically challenging due to complex genomes, polyploidization and an extensive historical nomenclature, potentially hiding cryptic taxa. A straightforward interpretation of phylogenetic relationships within the section *Mentha* is further hindered by dominant but outdated concepts on historically identified hybrid taxa. *Mentha spicata* is traditionally considered to be of hybrid origin, but the evidence for this is weak. Here, we aim to understand the phylogenetic relationships within the section *Mentha* using large sample sizes and to revisit the hybrid status and identity of *M. spicata*. We show that two of three traditional species in the subsection *Spicatae* are polyphyletic, as is the subsection as a whole, while the real number of cryptic species was underestimated. Compared to previous studies we present a fundamentally different phylogeny, with a basal split between *M. spicata* s.s. and *M. longifolia* s.s. Cluster analyses of morphological and genotypic data demonstrate that there is a dissociation between morphologically and genotypically defined groups of samples. We did not find any evidence that *M. spicata* is of hybrid origin, and we conclude its taxonomic status should be revised. The combination of genetic and phenotypic information is essential when evaluating hyperdiverse taxonomic groups.

## 1. Introduction

An important goal in evolutionary biology is to understand the evolutionary processes that promote biodiversity and taxonomic abundance [1]. Describing and analyzing phenotypic and genotypic variability is important to understand how variation is structured [2], which sheds light not only on the existing diversity but also on the genotype–phenotype links and their interactions with the environment. This includes aspects of polyploidy and interspecific hybridization [3], epigenetic factors [4] and plasticity, all essential in understanding the processes shaping biodiversity and speciation [5]. Central to understand the evolutionary process that shapes biodiversity is the concept of what constitutes a ‘species’ [6,7]. The classical species concept has largely been questioned in the genomic area, especially for plants. Populations of genetically structured species can sometimes be strongly divergent and, when put in a fragmented metapopulation context with incomplete reproductive isolation, specific assortments can often be cryptic [8,9,10,11,12]. However, DNA and species delimitation criteria have been successfully used for the detection of such species [13,14,15,16]. While molecular markers can be useful in deciphering complex relationships, they can sometimes be insufficient on their own (e.g., [17]).

Cryptic and hyperdiverse species complexes [6] with large distribution ranges are excellent model systems for studying the evolutionary processes shaping biodiversity as they harbor an abundance of genetic material for divergent selection and local adaptation. However, the genotype–phenotype links are not always clear in such taxa [18], and hence it can be difficult to describe their taxonomic structure [7]. With no clear concept of species delimitations, describing the genetic distance between populations and species in these groups can also be problematic. In addition, some hyperdiverse taxa show large amounts of gene flow, further complicating genetic groupings and taxonomic assignments [1,19].

Here, we use the genus *Mentha* (mints, subtribe Menthinae, family Lamiaceae; [20,21]), with a particular focus on the Spicate mint complex (subsect. *Spicatae* belonging to sect. *Mentha*, sensu Briquet [22]), to demonstrate the problems with taxonomic assignments and phylogenetic reconstructions in plant genera with large interspecific overlaps in phenotype and genotype. Further, this genus is known as a model for other polyploid taxa [23]. Moreover, taxonomic controversies have a long tradition in Lamiaceae, with the Menthinae being one of the most notoriously difficult groups [24], and therefore, a sound phylogenetic framework is needed [21].

Many authors (e.g., [25,26,27,28,29,30,31,32]) have already described the complex (morphological, chemical and molecular) diversity in mints. The complicated historical nomenclature (2630 names for *Mentha* on https://www.tropicos.org and https://www.catalogueoflife.org; accessed on 17 January 2021) strongly contrasts with the apparent simplicity presented by some authors nowadays. For example, Tucker and Naczi [33] list 18 species of *Mentha* and a high number of provisional subspecies. Additional species within the subsection *Spicatae* have also been described by, e.g., Borissova [34], Jamzad [35], Jančić [36] and Conn and Duval [37]. Despite the historical description of a large array of mint taxa (e.g., [38,39]), currently only five ‘basic’ species in *Mentha* sect. *Mentha* are recognized. In addition, numerous subspecies, varieties (mostly cultivars) and hybrids are also mentioned [33]. However, the historic richness in mint names likely reflects hyperdiversity and hidden cryptic species [40].

Spicate mints are currently divided into three basic species, *M. spicata* L., *M. longifolia* (L.) L. and *M. suaveolens* Ehrh. (nomenclature follows Tucker and Naczi [33] unless otherwise stated), with *M. spicata* commonly accepted as an ancient stabilized polyploid hybrid between the latter two species [41]. The idea of *M. spicata* as a hybrid species is linked to its well-promoted cultivation status [42,43,44,45] and to the generally accepted hypothesis that it behaves as a segmental allopolyploid with parental characters occasionally segregating [41,46,47,48,49,50,51]. Inspired by the work of Harley [48], Lebeau [52,53,54] described two subspecies of *M. spicata,* the ‘cultivated’ *M. spicata* subsp. *glabrata* (Lej. & Courtois) Lebeau, with glabrous leaves, and ‘the wild’ *M. spicata* subsp. *spicata*, with hairy leaves. However, their distinction as different subspecies is all but clear, and its taxonomic value has been questioned [55]. In addition, it seems that the taxonomic units *M. spicata* and even more so *M. × villosa* Huds. (and other authors) have sometimes been used as a taxonomic ‘garbage bin’. This has complicated phylogenetic, biogeographical and evolutionary studies. In particular, the phenotypic and especially the genotypic variation within and among the currently recognized mint species remains largely undescribed [56]. To resolve these issues, the integration of phenotypic and genotypic information of an increased number of individuals per taxonomic unit is needed, provided an appropriate experimental setting to avoid misleading associations between phenotypes and genotypes.

It is indeed highly relevant to explore more phenotypic features for *Mentha* sect. *Mentha* than has been done so far. Much of the past morphological research concentrated on a limited number of features (usually about 10–30; e.g., [31,57,58]), focused on variables that are not always feasible or evaluated morphology in unstandardized environments [59,60,61]. Very few studies have focused on more in-depth analyses of macro- and/or micromorphology, such as microstructure features in palynological studies [59] or different anatomical/microscopical features assessed in *M. suaveolens* [62]. Moreover, Bokić [63] measured many morphological and phytochemical variables (including stamen features with high intraspecific variability) for a large number of samples but did not perform genetic analyses. Other studies evaluated morphology in a standardized setting, but the applied genetic markers are meanwhile outdated (e.g., RAPD in [64]). In general, detailed morphological datasets from plants grown in standardized conditions are rare, and if present, they usually lack complementary molecular data (among the exceptions is [65]). Moreover, extensive studies concentrating on nuclear marker (ITS) information and considering at least three species of Spicate mints with sample sizes *n* > 20 are limited to just a very few (e.g., [66,67]). Remarkably in such cases, morphological data are lacking, and one must resort to plant labels projected on sequence dendrograms, giving way to dubious interpretations and vicious circles. Notwithstanding the lack of adequate and versatile information, the conclusion that *M. spicata* is an allotetraploid hybrid of the diploid species *M. longifolia* and *M. suaveolens* is repeatedly echoed, which nails the Spicate mint phylo-geny down to a simple triad with *M. spicata* in the middle (e.g., [33] pp. 8–9). To synthesize, based on existing data, the make-up of a detailed overview of the genotype–phenotype relationships within Spicate mints is highly problematic, which complicates the derivation of sound interpretations of phylogenetic relationships within the genus.

Here, we introduce a fundamentally different viewpoint on the evolutionary relationships among Spicate mints. We present an innovative phylogeny implying refreshing insights on previous hybridization concepts and cryptic taxa. By combining genetic and morphological data, we were able to discern cryptic mint species and uncover new taxonomic units. A strong phenotype pattern emerges that seems largely decoupled from any specific genetic input data but is nevertheless in line with the ITS-based phylogenetic backbone. We show evidence that the taxonomy and hybridization status of *M. spicata* should be reconsidered, thereby pointing to the danger of traditional concepts attached to outdated but inflated nomenclature so typical for cryptic taxa in general. With this study, we also illustrate the importance of simultaneously incorporating phenotypic and genotypic data of a large number of samples per taxonomic unit to understand the biodiversity and species richness in hyperdiverse taxonomic groups that likely hide a number of cryptic taxa.

## 2. Results

### 2.1. DNA Amplification, Phylogenetic Reconstruction and ITS Clusters

The amplification and Sanger sequencing of the ITS1-5.8S-ITS2 region was successful for all the samples, resulting in an alignment of 162 sequences (690 bp of which 167 sites are polymorphic). Some of the samples showed noisy chromatograms, but reamplification and resequencing with a more stringent protocol resulted in clear sequences for all but two samples. For sample 626, multiple peaks were observed that can be explained by at least four phased sequences (626a–d). Due to the noisiness of the chromatogram for sample 499, even with the more stringent protocol, only the major sequences were reported. For another twelve samples, the chromatograms indicate the presence of more than two sequences (Table S1), suggesting polyploidy or pseudogenes (gene duplications followed by mutations). However, only the two major sequences are reported here, as cloning would be needed to distinguish the minor sequence variants (peaks < 50% the size of the major peaks).

**Figure 1:**
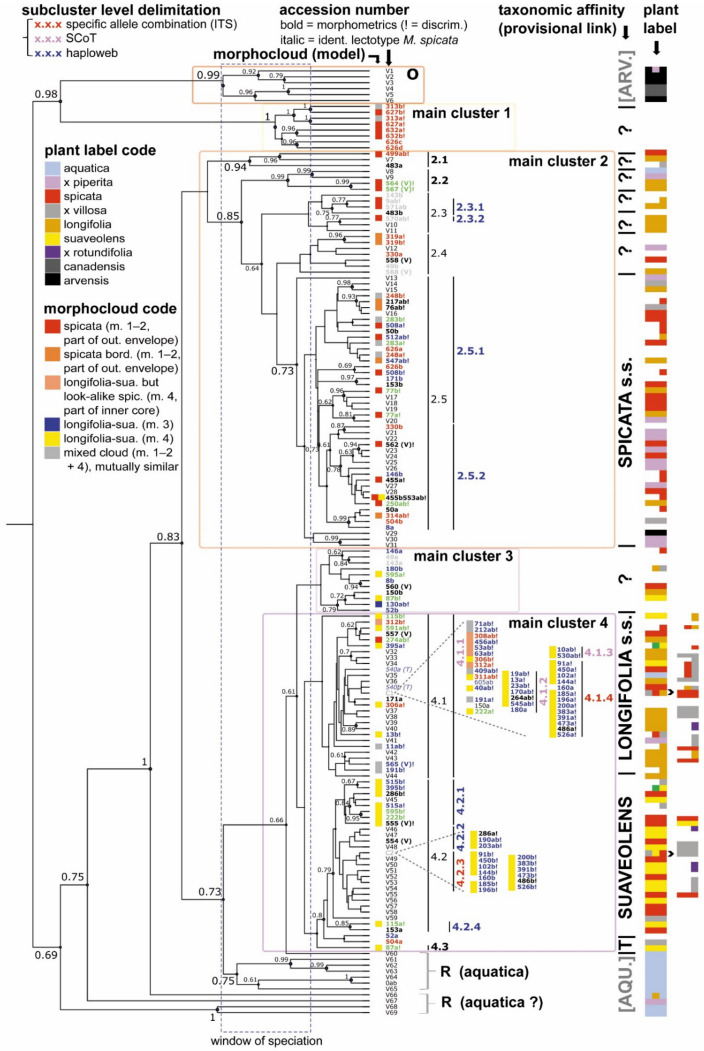
Ultrametric tree showing phylogenetic relationships within the genus *Mentha* subsect. *Spicatae.*

Although the Bayesian phylogeny shows some nodes with good support (posterior probability (PP) ≥ 0.70; [57]), most relationships are only moderately to poorly supported (Figure 1). There are, however, recognizable phylogenetic clusters that are taxonomically relevant (Figure 1). However, the basal split between what is interpreted by us as *M. longifolia sensu strictu* (cf. lectotype of *M. longifolia* (L.) L. [68]) and *M. suaveolens* is unsupported (Figure 1); notably, these are the only two Spicate taxa commonly considered as ‘pure’ species. Clearly, the traditionally recognized Spicate mint species are mostly polyphyletic (Figure 1; Table 1).

The ultrametric tree is based on a Bayesian analysis computed with BEAST. The phylogram mentions PP > 0.60 and main clusters, clusters and subclusters (subclusters based on haploweb, SCoT or a specific sequence combination in ITS). For currently existing taxonomic units, the window of speciation is shown. Further indicated are accession numbers (italic represents sample identical to the lectotype of *M. spicata* L.; bold covers samples with morphometrics, ‘!’ if included in DA; color refers to geographic origin for wild taxa: (a) Western Europe (blue), (b) Mediterranean area to Middle East including North Africa (green), (c) Eastern Europe to Eurasiatic (red), (d) Asia and South Africa (white), (e) unknown (black)), C1/C2, meaning different accessions with exactly the same sequence; morphocloud code; (new) taxonomic identity (provisional); and plant labels, with C1/C2 accessions in the margin (colored rectangles; for an extended list, see Table S1).

‘Taxonomic affinity’ links names to sequence clusters: cluster O, *M. arvensis–M. canadensis*; cluster 1, unknown Spicate mint species phylogenetically related to the species in cluster O; cluster 2, *M. spicata* s.s. and five different related species; cluster 3, an unknown cluster looking somewhat similar to *M. longifolia* s.s. but perhaps composed of mostly complex hybrid genomes; cluster 4, *M. longifolia* and *M. suaveolens* (different subspecies) plus *M. suaveolens* subsp. *timija* (‘T’); cluster R, *M. aquatica* (and hybrids such as *M.* × *dumetorum* belonging to R [aquatica ?]). Unknown taxa or complex hybrid branches are marked with ‘?’; i.e., this points to the presence of separate taxa that are either new taxa or taxa that might have (had) a historical name but that we do not know for sure (which one) so we do not consider as appropriate to mention.

The first phylogenetic division splits the sequences into two groups, one encompassing main clusters O and 1 and another encompassing all other samples (Figure 1). The main cluster O (PP = 0.99) groups sequences from *M. arvensis* L., *M. canadensis* L. and *M. × gracilis* Sole (*M. × gentilis* L.; Figure 1). These taxa have traditionally been classified in the taxonomic subsection ‘*Verticillatae’* [22].

**Figure 2:**
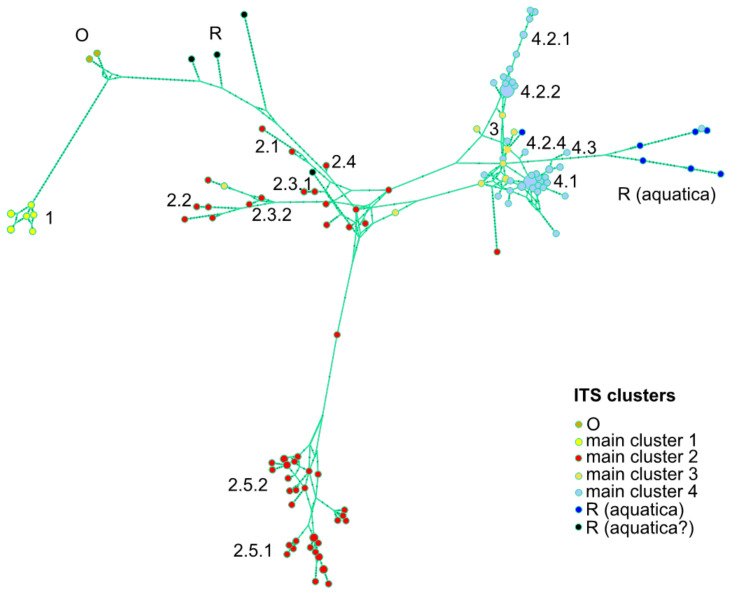
Haplotype network. Subcluster codes indicated next to their corresponding fields for recombinations. Colors according to ITS main clusters. Small black points indicate number of mutations compared to the closest sequence.

The main cluster 1 (PP = 1.00) encompasses a distinct group of sequences amplified from samples belonging to the classic phylogenetic subsection ‘*Spicatae*’ (Figure 1). These sequences are also found on an isolated branch in the haploweb (Figure 2). Most other samples traditionally grouped among the Spicate mints are found in the main clusters 2–4 (Figure 1). Basal to the main clusters 3–4 and to the main clusters 2–4 there are two groups of samples (‘R’) that are taxonomically recognized as *M. aquatica* or its hybrids *M.* × *piperita* or *M.* × *dumetorum*, which traditionally belong to the phylogenetic subsection *‘Capitatae’* [22] (Figure 1; Table 1).

The phylogenetic main cluster 2 contains sequences amplified from a diverse set of plants with labels including *M. spicata*, *M. longifolia* and *M. × piperita* (Table 1), and it can be further divided into five clusters (2.1–2.5; Figure 1). While labels of *M. longifolia* are also present, most specimens labeled *M. spicata* or *M.* × *piperita* found in this part of the phylogeny are monophyletic (2.5; Figure 1, Table 1), and here we define this as *M. spicata* sensu strictu. Using the haploweb, it is possible to further distinguish subclusters 2.3.1 and 2.3.2, corresponding to what is known as *M. longifolia* subsp. *hymalaiensis* (Briq.) Briq. and *M. longifolia* subsp. *capensis* (Thunb.) Briq, respectively, as well as subclusters 2.5.1 and 2.5.2 (Figure 2). Many of the sequences amplified from samples labeled as the hybrid *M. × piperita* are closely related to sequences from *M. spicata* s.s., in particular to those in subcluster 2.5.2 (Figure 1 and Figure 2), which might suggest a specific *M. spicata* s.s. lineage as one of the parents.

Main clusters 3 and 4 contain sequences amplified from a large range of taxonomically recognized species (Figure 1, Table 1). Samples found in cluster 3 morphologically resemble *M. longifolia* s.s. Sequences from this cluster are spread out across the haploweb, indicating a heterogeneous mix with hybrid properties (Figure 2). Although the relationships among the sequences within cluster 4 are mostly unsupported, some subclusters can be recognized (Figure 1). Cluster 4.1 contains genetically very similar sequences amplified from samples mostly labeled as *M. longifolia*, *M. × rotundifolia*, *M. spicata* and *M. × villosa* (Figure 1 and Figure 2). These sequences are highly similar to those amplified from an analog to the type specimen of *M. spicata* L. (sample 540; Figure 1 and Figure 2). Here, we identify this cluster as *M. longifolia* (L.) L. s.s., including subsp. *typhoides* Harley and subsp. *longifolia* with relatives.

Cluster 4.2 encompasses sequences amplified from samples labeled *M. suaveolens, M. × rotundifolia, M × villosa* and *M. spicata* (Figure 1, Table 1). This cluster is here identified as *M. suaveolens* and hybrids with *M. suaveolens*. Hence, some plants formerly labeled as *M. × villosa* or *M. spicata* (or sometimes even as *M. × cordifolia*) group with *M. suaveolens* s.l. Clade 4.2 is further split in the haploweb, with subcluster 4.2.2 corresponding to *M. suaveolens* subsp. *suaveolens* and subcluster 4.2.3 corresponding to *M. suaveolens* subsp. *insularis* (Figure 2). Sequences from sample 515, historically once referred to as *M. × billotiana* Déségl. & Durand, are included in subcluster 4.2.1, which mostly contains sequences from plants labeled *M. × villosa* or *M. spicata* (Figure 1 and Figure 2). Sequences from sample 286, a hybrid taxon known as ‘Moroccan mint’ (*M. spicata* var. *crispa* ‘Moroccan’, *M. × villosa* Huds. [51] or *M. spicata/M. crispa*), are found in subclusters 4.2.1 (*M. billotiana*) and 4.2.2 (*M. suaveolens* subsp*. suaveolens*; Figure 2). The single sample representing *M. suaveolens* subsp. *timija* (cluster 4.3) is found basal to cluster 4.2 (Figure 1; Table 1).

Sequences amplified from samples here identified as the hybrid *M. × rotundifolia* are found in two phylogenetic positions (Figure 1) as well as in two positions in the haploweb (Figure 2). These sequences are found in clusters 4.1, together with sequences amplified from *M. longifolia*, and 4.2.2, together with sequences amplified from *M. suaveolens* subsp. *suaveolens* (Figure 1 and Figure 2), which is consistent with *M.* × *rotundifolia* being a hybrid between *M. longifolia* (s.s.) and *M. suaveolens*.

In general, main cluster 2 can be recognized as the ‘*M. spicata* s.s. complex’, whereas main clusters 3 and 4 can be considered to represent the ‘*M. longifolia* s.s.–*suaveolens* complex’. Although *M. arvensis*, *M. canadensis* and *M. aquatica* can be considered as outgroups to the Spicate mints, we found that some of the sequences amplified from these species do indeed cluster within the Spicate mints; this shows that based on ITS sequencing, there is no exclusive division between formerly defined phylogenetic subsections sensu Briquet [22].

### 2.2. Start Codon Targeted Polymorphism Technique Integrated with ITS Sequences

The results from the SCoT analysis varied between markers; six markers (MPST2, MPST11, MPST14, MPST16, MPST18 and MPST30) presented amplifications for all samples, whereas four markers (MPST12, MPST13, MPST17 and MPST27) amplified only from a subset of samples. The absence of amplification is probably due to a lack of a particular marker, but primer site mutations cannot be ruled out.

**Figure 3:**
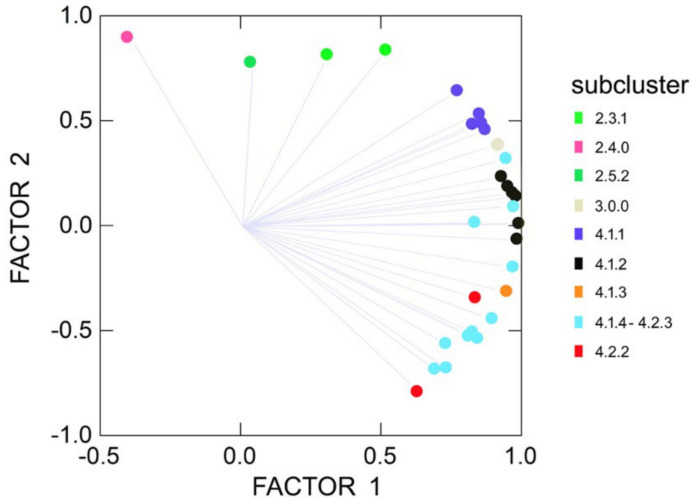
Plot of double factorized SCoT-ITS distances. Subcluster codes are indicated (for explanation, see text).

The first PCF axis (λ_1_ = 21.83; σ_1_^2^ = 68.2%) separates samples in subclusters 2.3.1, 2.4 and 2.5.2 from all other samples (Figure 3). The second dimension (λ_2_ = 7.36; σ_2_^2^ = 24.8%) then separates samples in the subclusters 3 and 4.1 (*M. longifolia* s.s.) from those of 4.2.2 (*M. suaveolens* subsp. *suaveolens*). Samples recognized as the hybrid *M. × rotundifolia* (4.1.4 × 4.2.3) are found between or intermingled with the two parental species *M. longifolia* (4.1) and *M. suaveolens* (4.2.2; Figure 1 and Figure 3). This taxon includes some samples traditionally labeled as *M. × villosa* var*. alopecuroides* (Hull) Briq., that consequently should be changed into *M. × rotundifolia* nothovar*. alopecuroides.* Nevertheless, *M. × rotundifolia* is considered as a separate unit here (4.1.4 *×* 4.2.3).

The SCoT analysis further revealed three clear groups of individuals within the phylogenetic cluster 4.1 (*M. longifolia* complex s.s.), subclusters 4.1.1, 4.1.2 and 4.1.3. The first two subclusters correspond to *M. longifolia* L. subsp. *longifolia* and the related *M. longifolia* subsp*. nemorosa* (Willd.) Wimm. & Grab./*M. nemorosa* Willd., respectively. Confusingly, samples found in subcluster 4.1.3 are traditionally typically labeled as *M. spicata* L. subsp. *spicata*.

Combining the results of the phylogenetic, haploweb and SCoT analyses, a total of 17 genotypic groups of samples is recognized that can be associated with specimen units (Table 1, Table S1).

### 2.3. Genetic Distances

Genetic distances show large differences depending on the specimen units considered (Table 2). The highest GD_min_ values are found between specimen units 1.0.0 and 2.2.0 or 4.2.2, and the lowest values are found between 4.1.4 × 4.2.3 and 4.2.1 (Table 2). There is a clear GD_min_ gradient from high to low values in Table 2, pointing to highly related units within the main clusters 3–4 (low values, distances ≤ 2.5%; see also Figure 1). The units 1.0.0, 2.1.0 and 2.2.0 appear to be relatively distinct (high GD_min_, distances range from 4.1% to 18.8% with few exceptions). The units 2.3.1, 2.3.2, 2.4.0, 2.5.1 and 2.5.2 are intermediate. A conservative estimate reveals at least nine different species plus several subspecies within the Spicate mints.

ABGD results (Table S1), including voucher specimens not included in morphological analyses or in the GD_min_ crosstable, suggest a potential range of 13–50 species (‘groups’) within Spicate mints, which is comparable to GMYC estimates (*n* = 18–51 including Capitate mints with a minimum of 13 for Spicate mints only). However, these values need to be confirmed using genetic and nongenetic data [70]. The lower limit confirms more or less the presented crosstable results with the exception that, surprisingly, *M. longifolia* s.s. (4.1) and *M. suaveolens* (pro parte; 4.2) are not grouped as separate species within the 13–50 species number range. We refer to the ‘window of speciation’ in Figure 1 for a visualization of the (relative) time frame where the main radiation of today’s taxa within sect. *Mentha* appeared. It is remarkable that a ‘final’ separation between *M. aquatica* and the majority of the Spicate mints starts at about the same time as a major radiation within the *M. arvensis* complex. Meanwhile, the Spicate mints, while mostly relatively young, had already formed some basic complexes, including remote clusters (e.g., 2.1).

### 2.4. Morphological Assessment of Genotypic Units

#### 2.4.1. Variable Selection

The subset of applied variables in Ward’s cluster analysis after correcting for multicollinearity is given in Table S2. A total of 21 variables (17.2%) did not enter Ward’s cluster analysis. These variables mainly concern (redundant) calyx variables or common generative features (*n* = 19) and a few vegetative features (*n* = 4).

**Figure 4:**
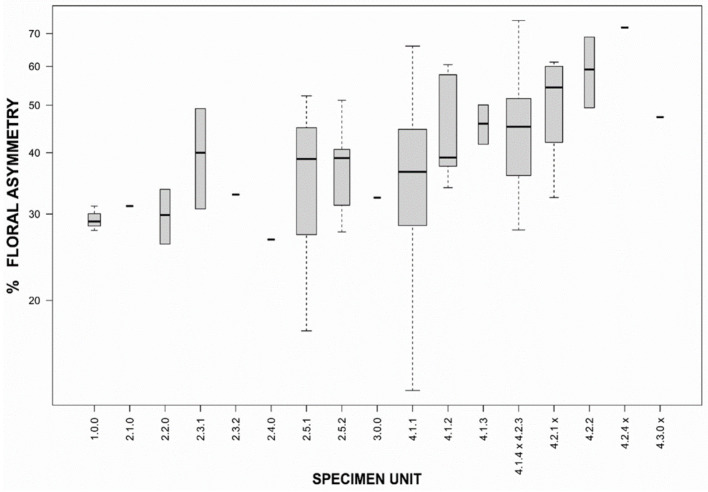
Boxplot: degree of floral asymmetry per specimen unit. Plot indicating the percentage asymmetry (the combination of the variables numbered as 27 and 32; Table S2) observed in flowers belonging to each of the studied genotypic units.

In DA, the preselection with lasso penalization (controlling for multicollinearity) was evaluated to be more fruitful with glmnet selection (λ = 0.98: axis_1_ dispersion = 57.5%, axis_1–3_ cumulative dispersion = 84.9%, Stewart–Love_1–6_ = 32.3%, R^2^ = 72.9%) compared to penalized LDA selection (λ = 0.20: axis_1_ dispersion = 40.9%, axis_1–3_ cumulative dispersion = 91.7%, Stewart–Love_1–6_ = 31.6%, R^2^ = 63.4%) because of a (slightly) higher representativity, great between-unit dispersion already at the main axis (although cumulatively over the dimensions a bit lower) and a strong systematic relationship over the genotypic units. The suitable λ-value was determined between the minimum and the minimal error value. This resulted in the best set of six variables (*n*_min_) that are equally divided among generative and vegetative features (Table S2): floral asymmetry (Figure 4) and flower color (*n* = 3), and leaf and stem features (*n* = 3).

#### 2.4.2. Unconstrained Morphology

A total of seven morphological Ward’s groups were identified by phenetic analysis (Table 1). Discriminant analysis (DA) was then performed on the 17 genotypic specimen units, and Ward’s groups were plotted (Figure S1). There is a limited association (λ = 0.30) between the Ward’s groups and the specimen units and only a partial significance between the Ward’s groups and the two most important DA axes (Kruskal–Wallis *p*_1_ = 0.000, *p*_2_ = 0.394). This indicates that the Ward’s projected groups (morphotypes; Figure S1) harbor high genetic heterogeneity and show only partial and limited correspondence to the genotypically induced DA-space (with a distinction along the first axis suggesting a primarily one-dimensional, hence one-sided, interpretation in terms of morphology; see below). There is, however, a very obvious correspondence between the unconstrained morphological Ward’s groups and the plant labels (Table 1), which is somewhat expected given that mint taxonomy is currently largely based on morphology. Thus, traditionally groups are (superficially) perceived as (1) *M. spicata* (subsp. *glabrata*) or as *M. × cordifolia*; (2(−1)) *M. longifolia* including *M. asiatica*, *M. longifolia* subsp. *hymalaiensis*, *M. longifolia* subsp. *capensis*; (3(−2)) *M. longifolia* or *M. spicata* subsp. *tomentosa*/subsp. *condensata*/*M. microphylla*; (4) *M. longifolia* or hybrid; (4–5) *M. spicata* subsp. *spicata* or *M. × villosa*; (6) *M. suaveolens* (subsp. *suaveolens*) or its hybrids, including *M. × villosa* (subsp. *alopecuroides*) and *M. × rotundifolia*; (7a) *M. suaveolens* subsp. *insularis* or *M. timija* plus *M. suaveolens* (subsp. *suavolens*) or *M. × rotundifolia*; and (7b) *M. spicata* (subsp. *spicata*). The genetic heterogeneity within these Ward’s groups is clear from Table 1 when inspecting the genetic (sub)clusters.

#### 2.4.3. Constrained Morphology

**Figure 5:**
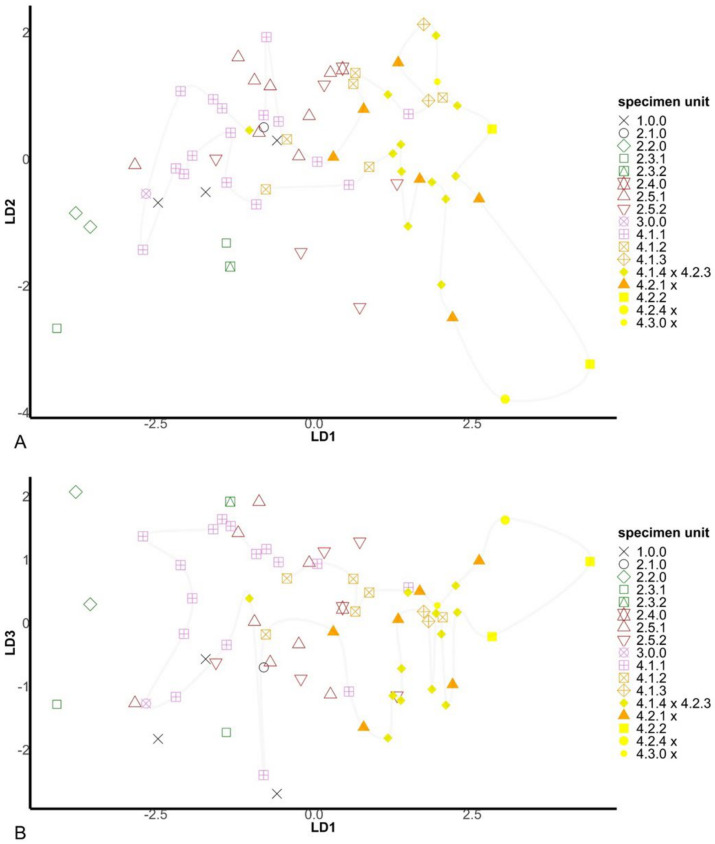
Discriminant analysis relating ITS-based genotype to specimen units. Symbols and colors according to genotypic specimen units (based on observed genotypes). Light grey polygons are manually drawn to indicate the outer perimeter of the *Mentha longifolia* s.s.–*Mentha suaveolens* complex, as a central core within the *Mentha spicata* s.s. complex (outer shell). (**A**) DA1–2. (**B**) DA1–3.

**Figure 6:**
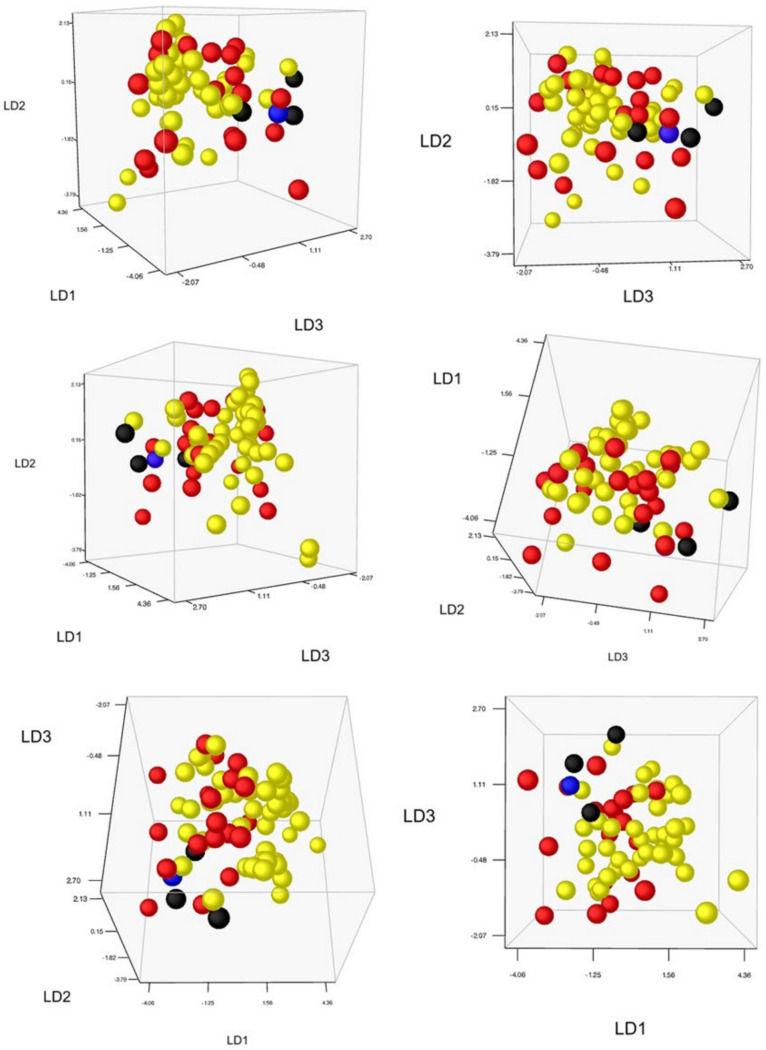
Discriminant analysis visualizing the general morphocloud pattern. Discriminant analysis results with 3D pattern reflecting general relationships between main groups. The inner core–outer envelope shows how the *M. longifolia* s.s.–*suaveolens* complex and the *M. spicata* complex, respectively, are mutually connected and at the same time also largely separated. The outer envelope contains accessions with sequences belonging to main clusters 1 (black) and 2 (red), while the inner core has sequences belonging to main cluster 4 (yellow). Note that the only morphologically analyzed accession with sequences from main cluster 3 (blue) is ordinated at the outer edge.

The Stewart–Love canonical redundancy index equals 0.323 when based on the first six dimensions of the constrained DA-space as well as the full morphospace (a PCF with a cumulative six-dimensional variance of 46.0% of the total morphological variation). This implies that 32.3% of the generic signals of the full morphospace are explained by the variation among the specimen units in the reduced (constrained) morphospace. However, 96.3% (Δ_W_ = 0.037, *p* < 0.001) of the between-group morphospace dispersion among the 17 genotypically defined specimen units is explained by the six significant morphological variables (Figure 5 and Figure 6, Table S2).

**Figure 7:**
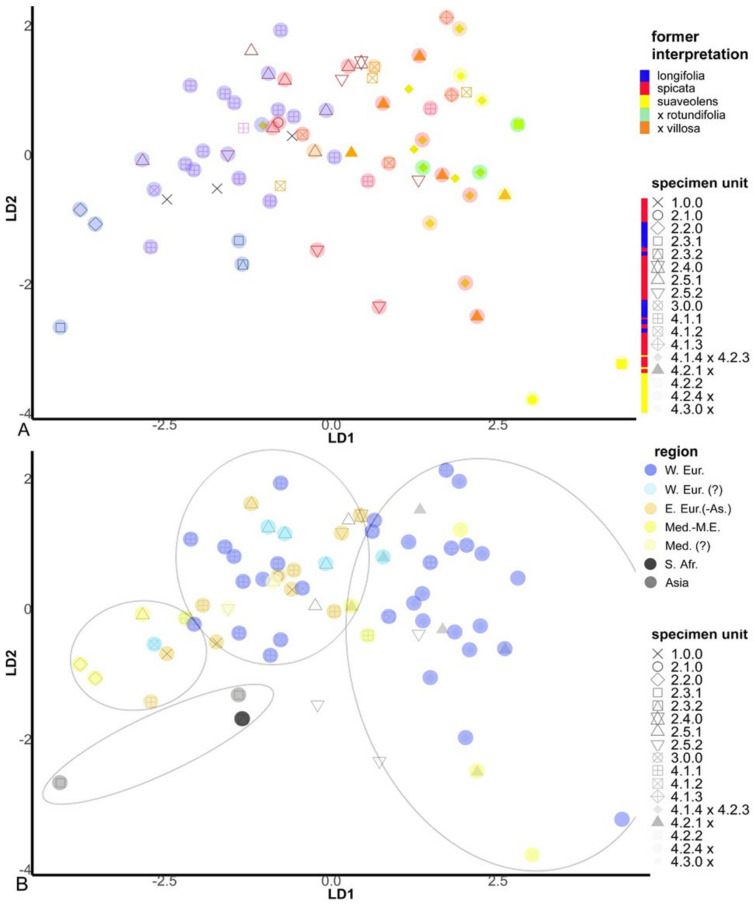
Species perceptions and mint biogeographical origin plotted on discriminant analysis results. Exogene data plotted on existing discriminant analysis. Symbols according to genotypic specimen units. (**A**) Former species interpretation plotted on DA1–2. Colors represent ‘classic’ species (based on plant labels). (**B**) Mint biogeographical regions plotted on DA1–2: (a) Western Europe; (b) Mediterranean area to Middle East, including North Africa; (c) Eastern Europe (to Eurasiatic); (d) Asia; (e) South Africa. Colors represent different regions of origin from the wild gene source. Symbols in grey (no color) are cultivated or introduced taxa without known origin/location. Ellipses are arbitrary as manually added.

What has historically been perceived as *M. longifolia* (s.l.) (cf. Figure 1, clusters 2.1, 2.2, 2.3 and 2.4) is found on the negative side of the first axis, and the classic *M. spicata* (s.l.) (including *M.* × *villosa*) is mostly found at the positive side of the first axis (Figure 7A). However, *M. spicata* s.s. as defined here (cf. cluster 2.5) is mainly situated central on the first axis, bordering and partly intermingling with *M. longifolia* s.s. (4.1.1, 4.1.2; Figure 6A). Importantly, DA demonstrates (Figure 5 and Figure 6) an emerging pattern among the specimen units that gives the impression of an ‘inner core’ of the *M. longifolia* s.s.−*suaveolens* complex and an ‘outer envelope’ of *M. spicata* s.s. with relatives and unit 1.0.0. It shows that there is only limited morphological overlap between accessions with sequences belonging to the different main phylogenetic clusters (Figure 6). This is even more clear after DA-morphocloud clustering (Figure 1 and Figure S2). This cluster analysis result contains a very limited number of ‘aberrant classifications’, i.e., accessions genetically belonging to one main cluster but morphologically to another main cluster (2.8% are aberrant when excluding 7.1% borderline cases and 7.1% *M. spicata* s.s. ‘look-alikes’ that are actually *M. longifolia* s.s. residing in the ‘inner core’ morphocloud; Figure 5). Some of the accessions (12.9%) do, however, occur in mixed morphoclouds where plants from the *M. spicata* s.s. complex morphologically resemble plants of the *M. longifolia* s.s.–*suaveolens* complex. Hence, a total proportion of 84.3% of the analyzed accessions show a morphology that is unambiguous with respect to their phylogenetic relationships.

Interestingly, the first two axes of the DA plot also reflect a morphological distinction between West European and Mediterranean (including Northwest African) *M. suaveolens* and associates (units 4.2(.1–2)/4.2.4× and 4.3 including hybrids) and the geographically more remote mints from Asia and South Africa (units 2.2 and 2.3(.1–2); Figure 1 and Figure 7B; Table S1). At the extreme, Southeast European and Eurasiatic mints form a cloud (Figure 7B). All other mints, however different from each other but usually pan-European, are squeezed in between (Figure 7B). Mints originating mostly from other continents outside direct European influence (2.3.1, 2.3.2) and some Southeastern European mints (2.1.0, 2.2.0) are unfolded in the DA1–3 plane but barely intermingle with *M. longifolia* s.s. or with other taxa except Eurasiatic mints (1.0.0; Figure 7B).

## 3. Discussion

### 3.1. A Revised Phylogeny

Here, we present an inter- and intraspecific evaluation of both genotype (ITS + SCoT) and phenotype within the genus *Mentha,* resulting in a revised phylogeny of the Spicate mints. To our knowledge, this evaluation is the most comprehensive of its kind. Our findings suggest that the Spicate mints are polyphyletic (main clusters 1–4, Figure 1) and much more diverse than previously thought. The obtained ITS GD values suggest at least nine species among the analyzed samples (Table 2), an estimate that is supported by the lower limit (*n* = 13) of the ABGD/GMYC analyses. However, the upper limits of the ABGD/GMYC analyses including vouchers suggest a large number of hidden (cryptic) species. Indeed, we were able to identify several cryptic taxa, e.g., *M. nemorosa* or *M. billotiana*, and diverse unnamed taxa (Figure 1 and Figure 2). While most of the traditional Spicate mints appear polyphyletic, we find a number of ITS clusters that can be interpreted as (redefined) *M. spicata* s.s., *M. longifolia* s.s. and *M. suaveolens* s.l. (Figure 1). Obviously, the distinguished genetic subclusters globally correspond to a set of recognizable phenotypes described by a low number of relevant variables (e.g., Figure 4). In summary, we present a fundamentally different phylogeny compared to previous publications, with totally remote Spicate mint taxa and a basal split between *M. spicata* s.s. and *M. longifolia* s.s., also questioning the identity of the lectotype of *M. spicata*.

The DA results offer a nice arrangement of genotypic units sorted along principal dimensions, pointing to a substantively strong correspondence between the modeled morphology and the genetic groups at a higher taxonomic level (Figure 6). The overwhelmingly consistent inner core–outer envelope pattern emerged irrespective of the imposed genotypic structure, which highlights the relevance of the selected morphological variables regardless of technical and statistical qualities in a model context that initially led to the (penalized) selection. Therefore it is probably no coincidence that exactly highly relevant morphological features at genus or family level such as floral asymmetry (Table S2, Figure 4) show systematic and meaningfully structured differences for varying genotypic groups. Hedge [71] pointed to *Mentha* as an example of evolutionary trends in the Lamiaceae family, not in the least concerning floral asymmetry (actinomorphic versus zygomorphic flowers). Traits such as floral asymmetry and varying patterns of oil glands are possibly involved in insect–flower coevolution [72,73,74,75,76], and such traits are likely to be important for speciation. Some of the selected leaf characteristics (such as petiole and venation; Table S2) also have ecological relevance, for example, in response to varying humidity levels in the atmosphere and soil or in relation to herbivory (e.g., [77]), and are linked to differential adaptation (e.g., [78,79]). The genetic and morphological distinctions between the species are further supported by obvious biogeographic differences between the taxa (Figure 7B).

As is discussed in the following, the current mint phylogeny still looks too much like a hotchpot; it straitjackets Spicate mints by their three recognized classic species, thereby dumping idiosyncrasies in a bottomless hybrid container. The Ward’s groups we present offer, via morpho-typology (Table 1), a plausible explanation for the classic taxonomic assignments within *Mentha.* However, as many of the traditionally defined taxonomic units are polyphyletic, a revised taxonomy taking into consideration both morphology and genetics is needed. Here we present a much-refined phylogeny with recognizable groups of samples that are also morphologically similar. In particular, we show that the Spicate mint phylogeny is characterized by polyphyly of previously recognized species and that its diversity is much higher than formerly conceived. This shows that much more intensive sampling is needed to fully understand the diversity gradients, elucidate cryptic taxa and define new taxa in *Mentha*. We also demonstrate that phylogenetically defined groups in this hyperdiverse genus can also be morphologically distinguished. Nevertheless, more research on, e.g., microstructures or ontogenetic features, not assessed here, might help in further elucidating phylogenetic relationships among the Spicate and Capitate mints. However, we do note that the morphospaces of many classical taxonomic units of Spicate mints are largely overlapping. Hence, taxonomic identification solely based on morphology is likely to remain problematic.

### 3.2. Former Phylogenies in the Light of New Results

Over the last couple of decades, there have been numerous phylogenetic studies of the genus *Mentha*, mostly of the section *Mentha,* using morphological, cytological and chemical characters (e.g., [59,62,63,80,81,82,83,84,85,86,87,88,89,90,91]) as well as molecular markers (e.g., [55,60,61,64,66,92,93,94,95,96,97,98,99,100,101,102,103,104,105]). However, despite the enormous amount of data gathered, the classic taxonomic divisions (e.g., [22,106]) still largely stand. Studies describing an integral phylogeny of the whole genus using molecular markers concluded that the Spicate mints are monophyletic [33,93,107]. However, most studies (but see [55,66]) have only investigated a limited number of species and/or individuals, largely ignoring inter- and intraspecific variation or discarding the taxonomical implications it has. Moreover, integrated genotype–phenotype analyses are not frequent, and often an insufficient number of genetic markers are used, or the morphological descriptors are scarce (but see [32,65]).

Molecular phylogenetic and phenetic studies have become most popular in the last two decennia and are mostly based on sequencing information (nrDNA or cpDNA) or on different marker methods (e.g., RAPD, ISSR and SCoT). An important phylogeny of the whole genus is, however, based on morphological data [33], and at least for sect. *Mentha*, the classic species remain: *M. longifolia* clusters together with *M. spicata* and is consecutively related to *M. suaveolens* (a generalized comparative scheme is given in Figure S3). Those taxa are considered as a tight monophyletic (Spicate mint) complex most closely related to *M. aquatica* and more distantly related to *M. arvensis*. Similar results have also been derived by different molecular techniques (for typical examples, see [108]), but also several different dissonant phylogenies exist as discussed further. However, the classic species (names) are not questioned, as they are always recycled. As a result, dissonance might be just an artifact of Babylonian labeling. Hence, diving into published Spicate mint phylogenetics, it is unfortunately hard to determine the difference between labeling effects and (true) genetic differences for certain when sequence data are scarce or unavailable. Gobert et al. [55] assessed relationships (by AFLP-based molecular marker technique) within sect. *Mentha* using many Spicate mints and concluded, at least as far as nrDNA data are considered, that *M. spicata* is intermediate between *M. suaveolens* and *M. longifolia*, with *M. aquatica* and *M. arvensis* together as a separate clade. According to Bunsawatt et al. [93] (ITS), *M. suaveolens* and sister species *M. spicata* form one single clade with *M. longifolia* and a ‘strain’ of *M. canadensis* (supposed to be a tertiary relict amphidiploid descending from *M. longifolia* × *arvensis* [109]), while *M. aquatica* forms a different clade, as does *M. arvensis* (with another strain of *M. canadensis*).

Gobert et al. [66] returned with a more elaborated ITS phylogeny (with accompanying cpDNA phylogeny) that has a basic resemblance to our phylogeny; however, due to the presence of confusing labels and different tree swapping, the molecular response looks quite noisy and the interpretation is very different (and polyphyletic) [66]. Moreover, the phylogeny contradicts former research results or at least suggests profound changes [55]. Although the name *M. spicata* is overly represented, the authors only included just one (two?) *M. spicata* s.s. plants (as defined here), which turns any direct interpretation of the tree into a nontrivial exercise [66]. To make things clear, we reanalyzed a number of their voucher sequences [66] (Figure 1 and Figure S3, Table S1), making a comparison with our results possible. Our phylogeny (Figure 1) is much more extended because of a higher sample size, with different remote groups, and our interpretation is very different. Nevertheless, the dendrogram given by Gobert et al. [66] reflects some but not all of the basic splits presented by us: the split between *M. arvensis*, followed by *M. aquatica* and finally the separation of the *M. spicata* complex from the *M. suaveolens–longifolia* complex, although Gobert et al. [66] did not interpret it that way. Using cpDNA (*trnL-trnF*), Bunsawatt et al. [93] concluded that ‘the seven species’ from sect. *Mentha* are not monophyletic. *M. aquatica* and *M. arvensis* (with *M. canadensis* and the associated *M. japonica*) form one clade (Figure S4) beside *M. suaveolens* on the one hand and *M. longifolia* with *M. spicata* on the other hand. According to Bunsawatt et al. [93], the sister group relationship of the latter two species suggests that *M. longifolia*, rather than *M. suaveolens*, is the maternal parent of *M. spicata* (as the *trnL-trnF* region is presumably maternally inherited). Interestingly, the considered ‘*M. longifolia*’ is, however, voucher specimen 564(V) (Figure 1) that is grouped in our phylogeny under cluster 2.2 (which is related to cluster 2.5, *M. spicata* s.s.; Figure S4). Thus, a low sample size is probably another reason why the historic interpretations of derived phylogenies remained ambiguous. Even the cpDNA phylogeny of Gobert et al. [66] is plagued by a lack of samples, but it still is the largest published cpDNA tree for Spicate mints ever. Most other relevant cpDNA phylogenies [25,93,97,107], as far as Spicate mints concern, more or less fit within the larger cpDNA phylogeny [66] but exceptions exist (see below).

The mentioned cpDNA phylogeny (Figure S4) reflects a series of splits where the highly diverse ‘*M. aquatica*’ plays the leading role (present in all the major branches). Al-though *M. aquatica* is not commonly interpreted as a hybrid species, it has a high ploidy level (mainly octoploid but also nonaploid [104]), and hence its polyphyly in the ITS-based phylogeny (Figure 1 and Figure S4) can possibly be explained by a high number of gene duplications with different evolutionary histories. The cpDNA phylogeny is, however, not as informative as our ITS phylogeny as the majority of the relationships within the Spicate mints remain unresolved (see also [25]). Contrary to all the former cpDNA results, Thakur [110] puts *M. arvensis* with *M. spicata* next to *M. aquatica*. We refer to the huge diversity of Spicate mints to interpret such dissonant results. Kalfagianni [67] (*trnH-psbA* and ITS) points again to strongly heterogeneous results signifying probably high putative intraspecific variability, but possibly due to labeling issues the results may be incomparable and inconclusive. Moreover, the *rbcL*-based phylogeny by Ahmed [28] is clearly different, which might also be a consequence of sampling (bias).

High intraspecific variability is clear from other molecular studies too (e.g., marker techniques AFLP, RAPD, ISSR and SCoT), which mostly led to very heterogeneous phylogenetic or phenetic results [32,61,98,104]. Choupani et al. [111] repeat for Spicate and Capitate mints (*M. longifolia, M. spicata, M. aquatica* etc.) the remarkable conclusion that intraspecific variability is larger than interspecific variability—a thesis that makes sense in a polyphyletic constellation where cryptic species might hide [40] (see also many other mutually deviant results as for interspecific relationships and variability in Spicate/Capitate mint species [30,31,60,112,113,114,115]).

As rightly summarized by Soilhi et al. [32], previous studies that combined molecular markers with morphological data found no agreement between the (clustered) results of both molecular and morphological markers separately [31,61,116]. Unfortunately, no such nrDNA or cpDNA sequence-based comparisons have been published in the case of Spicate mints. Only very few studies analyzed standardized morphological data with a high number of descriptors as we do (as far as we know, only [32,117], but the last one does not concern Spicate mints). So far researchers studied a very limited number of unstandardized morphological features that, moreover, show high intraindividual and intraspecific variability (e.g., pistil length, stamen length and their ratio), while at the same time genotypes of species (*M. spicata–M. longifolia*) cannot be separated in cluster results based on ISSR molecular markers [112]. The dendrogram presented by Soilhi et al. [32] represents morphotypes that yield, among other findings, a deep split within ‘*M. spicata’*, while the molecular data results show very different, totally mixed-up groups (where *M. longifolia*, *M. spicata*, *M. crispa* and *M. aquatica* or *M. × piperita* are repeatedly included). Common reasons given for discongruity include the character or genetic marker selections, the complex hybridization histories and phenotypic plasticity [32]. However, the idea of an unsuitable taxonomy and nomenclature never pops up. Although it is clear that there is high intraspecific variability, we suggest that this is partly caused by the historic complex taxonomy and that the classically used plant labels cause much of the current phylogenetic confusion within *Mentha*. We, therefore, suggest that a revised taxonomy is in order, one that takes both morphological and genetic data into account.

### 3.3. Perspectives in Mint Genetics

#### 3.3.1. Nuclear DNA versus Chloroplast DNA

ITS sequences are generally considered to be a relatively good phylogenetic marker to distinguish biological variability for plants [69]. Although several studies suggest that the most accurate phylogenetic reconstructions are obtained when multiple loci are combined, ideally targeting both chloroplast (*matK, ndhF, rbcL, rps16* and *trnL-F*) and nuclear markers (ITS) [118,119,120], it has been shown in Lamiaceae that one marker (e.g., *trnH-psbA*) can nicely resolve taxonomy [121]. Nuclear markers on their own appear to be promising tools to disentangle complexes of hybrids such as those found in the genus *Mentha*. Thus, we generated a global view on the genetic diversity and relationships within *Mentha* using SCoT in combination with the ITS marker. Some authors have argued that the use of the ITS marker in detecting cryptic species is limited because the evolutionary rate of the ITS region is too slow [122]. This appears to be the case for mints, where indeed supplementary analyses (Figure 2) and markers (Figure 3) are needed to refine clusters of individuals and sequences. Future comparisons should include genetic information from plastid markers, such as *trnH-psbA* and *matK*. While this may provide a better insight into, for example, relationships between seed and pollen dispersals, plastid marker data on their own will not suffice to generate a full phylogenetic overview given the importance of hybridization and polyploidy in *Mentha*.

Thus far, cpDNA phylogenies (Figure S4) appear to provide less refined phylogenetic relationships among mints compared to ITS-based phylogenies. However, cpDNA does add information on maternal lines and it does confirm a few of the basal splits also found using ITS data (Figures S3 and S4). The incongruences between evolutionary histories of nuclear and plastid DNA in the subtribe Menthinae are not surprising given the high frequency of interspecific hybridizations [123]. The low genetic differences found between mints using plastid markers [28,93,97,121,123] show that these markers on their own are not as good as nuclear markers to infer interspecific relationships between mints. Bunsawatt et al. [93] explicitly mention that some parts of the *trnL-trnF* and the ITS phylogenies lack resolution. When comparing the *trnL-trnF* spacer with ITS1-ITS2 in terms of percentage of variable nucleotide sites [93], the *trnL-trnF* region sequences are found to be about four times less variable (12% versus 47–51%).

Previous studies have suggested that the ITS sequence might show variation that is less useful to disentangle interspecific relationships [124] or that might confound the relationships between mints [93]. However, here we show that when combined with other markers, such as SCoT and morphology, ITS suffices to disentangle most phylogenetic relationships and that previous Babylonian confusion on phylogenies have been enhanced by the use of plant labels. In contrast, Figure 1 shows how a multidimensional response set of morphological markers is able to propose a meaningful composition that casts light on a complex phylogeny, without the need for any a priori taxonomic classification.

Combining cpDNA and nrDNA would help in future research to uncover both old and recent hybridization or polyploidization events on representative interspecific samples of natural populations, and using single-copy nuclear genes would also help with this endeavor [125,126]. However, to unequivocally resolve hybridization history, whole-genome sequencing of multiple individuals is required, and genetic admixture analysis [127] using a large number of single nucleotide polymorphisms (SNPs). Moreover, network-based representations would be more suitable to visualize the reticulate relationships present in mints, while there is a need for detailed population genetic studies based on samples over large biogeographic regions covering the diversity gradients.

#### 3.3.2. Intraindividual ITS Variability as A Mirror of Intraspecific Variation

The incongruence of different phylogenetic trees, particularly the differences between cpDNA and nrDNA, may hold in a nonconcerted evolutionary context with effects of recombination, hybridization or incomplete lineage sorting. Therefore, phylogenetic reconstruction of a genus overwhelmed by hybridization and polyploidization, such as *Mentha*, is challenging. Additional genetic challenges include intraindividual ITS polymorphism (IIP) and pseudogenes [127,128,129]. IIP is common [130,131,132,133,134,135] and can be structured in very different ways, but most often the interindividual variability is smaller than IIP variation (e.g., [136]). IIP can be due to, for example, incomplete lineage sorting, divergent and unconcerted paralogs, incomplete concerted evolution after hybridization, pseudogenization following gene duplications or recombination between ITS copies [124,133,134,137], all of which might influence the inferred phylogenetic relationships. In addition, the parent-biased homogenization of ITS following polyploidization or hybridization will affect the inferred phylogenetic tree and, hence, the derived evolutionary relationships. As a consequence, some authors advise against the use of ITS for phylogenetic inferences [137,138,139]. However, ITS is still a commonly used marker for phylogenetic studies of plants, and even in cases of abundant IIP, it has been shown that this marker is still useful for species identification (e.g., [140]). In ‘Better the devil you know?’ [141], the use of ITS is further recommended but strong warnings are also given. Thus, handling IIP as an extra source of information in *Mentha* implies the use of other concepts and, hence, distinct models, which turns IIP into a model challenge instead of a data problem. For instance, IIP can be an interesting tool for uncovering incomplete lineage sorting, introgression, hybridization and reticulation [138]. Indeed, the Bayesian method used here can deliver evidence of past hybridization and reticulate evolution [133]. The higher sequence variability generated by IIP both provides higher interspecific resolution and is associated with a higher intraspecific variability [140,142,143], which can be useful for identifying cryptic species [144,145]. The positive correlation between IIP and inter-/intraspecific variability generates patterns from which GMYC-based methods [146] can benefit in detecting cryptic taxa [144]. We also took GMYC principles into account (waiting times as a window of speciation on the ultrametric tree, Figure 1) and used them to support the estimation of species numbers within Spicate mints.

Disentangling alleles or phased sequence variations from nuclear markers using traditional sequencing techniques (i.e., Sanger sequences) often requires cloning, especially for species with high ploidy [147]. For relatively low frequencies of IIP, direct sequencing poses no problem [127], and here we did not find it necessary to use cloning to disentangle intraindividual sequence variants. Indeed, the nrDNA, including the ITS region, is often represented by hundreds of copies that in principle follow concerted evolution. Therefore, no more than one or two sequence types are expected to be observed in each specimen. Although the homogenizing effect of interlocus concerted evolution of paralogs can hide the history of one or more parental genomes in allopolyploids when standard PCR methods are used alone, this is not systematically the case [148]. Here, we find that only a minority of samples (8%) showed minor peaks (<50% the size of major peaks), and these samples are spread across the phylogeny (Figure 1 and Table S1) without being obviously clustered, except some samples found in main cluster 3. That clade largely consists of hybrid accessions. Only one sample (626, main clusters 1–2, Figure 1) showed more than two similar-sized peaks (Table S1). Two of the sequences (626c–d; main cluster 1) have a low GC content, which in principle could indicate pseudogenization, although no such cases were observed. This particular sample was also removed from the genotype–phenotype analysis. In conclusion, we do not believe that IIP had a negative interference on the presented phylogeny or on the integrated genotype–phenotype analysis. Indeed, in the integrated genotypic–phenotypic analyses, most of the hybrid taxa were not included.

### 3.4. Plasticity in Porous Genomes—Future Challenges in Mentha

The phylogenetic complexity within the genus *Mentha* mirrors the complications with species concepts in general, especially for taxa with porous genomes where introgression, hybridization and polyploidization have significant evolutionary impacts [18,149,150,151]. Hybridization and polyploidy have indeed most likely played important roles during speciation in mints, which forms one reason the number of taxonomically valid species is a subject of controversy [152,153]. The complex genomic networks of taxa with porous genomes cause phenotypic mosaics that behave dynamically [154]. Indeed, plasticity is known to be widespread in cryptic taxa such as *Mentha* (e.g., [155,156,157,158]), which confounds morphological identification. However, low levels of plasticity have been reported for *M.* × *verticillata* [159]. We cannot totally exclude the effects of plasticity here, but our garden setup would eliminate most of its side effects, including heteroblasty. Furthermore, the unexplained part of the morphological variation is likely linked to genotypic differences between taxonomic units that were not captured by the neutral markers analyzed here. Given the complex evolutionary dynamics of *Mentha,* including convergence, reticulation, introgression and incomplete lineage sorting (e.g., [151,160]), there is much reason to conclude that there is large-scale morphological variation in several clades of *Mentha*, making a clear general link of the full morphospace at the interspecies level unlikely to be found.

We stress that underestimations of the genetic and morphological diversity of mints can further bias sampling design and insufficient sampling efforts can indeed lead to spurious conclusions. Continued sampling of the same hybrids, varieties (cultivars) or special morphologies (as is the case with some herbarium specimens) may not shed new light on the overall evolutionary relationships within a genus, and may even generate flaws. According to Phillips et al. [161], in general, sample sizes of 15 to 25 (or 60) individuals per species are to be considered an absolute minimum level to obtain reliable estimates of genetic polymorphism at the species level, although it will depend on the species at hand [161,162,163,164]. However, the limited availability of funds, time and material frequently prevent such extensive sampling; hence, conclusions based on a handful of samples are the norm, although this serious caveat should be acknowledged, including from a statistical point of view.

Adding to the obvious complexity in genomic relationships and morphological overlaps between different mints, species assignment within this group of plants has been greatly complicated and confused by the historic description of a huge number of species, many of which are synonyms or based on a single individual with an unusual phenotype (e.g., [33]). The unrestrained proliferation of thousands of names (e.g., [38,39]) led to nomenclatural complicatedness, and as the tower of Babylon was built, perpetual and quasi-inherent confusion seemed to guarantee a persistent puzzling taxonomy. Chanting Babylonian plant labels without real-life metrics exerted on a standardized phenotypic space and without handling a sufficient sample size is counterproductive in phylogenetic research on cryptic taxa. Nomenclature is by nature hierarchical, but hyperdiversity breaks the limits of traditional species delimitations and makes classic nomenclature unfeasible. Hence, binomial nomenclature might not be able to offer an adequate solution on the species level as mints might indeed be genetically built multiway due to complex evolutionary relationships. Thus, *Mentha* is a classical example where the desire for a hierarchical and binomial nomenclature to fit a multidimensionally structured network of organisms complicates the understanding of the evolutionary history of a whole genus.

### 3.5. The Hybrid Status of Mentha spicata Revisited

*M. spicata* is commonly accepted to have a hybrid origin and is thought to have arisen as an ancient stable allopolyploid between *M. longifolia* and *M. suaveolens* [41,47,55,63,66,112,165,166]. It is, however, different from the equivalently defined hybrid *M. × rotundifolia*, which is often mistaken for *M. spicata*. Thereby, it is hypothesized that *M. spicata* probably arose in cultivation [49], which might have been in Neolithic times, i.e., roughly about 10–11 Ka BCE. However, viewed from a historical perspective, all of this could be read as a persistent myth in which the many-headed dragon ‘*M. spicata’* was gradually embedded—a myth that became shaped along misty pathways (e.g., [41,45,47,106]) and that is still very much alive today (e.g., [63,66,112,166]). Conclusions from repeated studies of morphology, chemistry and cytology, coupled with field experiments and genetic research, confirmed or enhanced the idea of such hybrid origin. In particular a genetically and morphologically intermediary position of ‘*M. spicata*’ (Figure 1 and Figure 6) has often been (mis)interpreted as a clear sign of hybridization (e.g., [41,55]). The intermediate position between *M. longifolia* s.l. and *M. suaveolens*, also inspired by too many haloed plant labels clumsily fixed to sequence phylograms, has been implicitly framed as a unidirectional relationship with ‘the two parents’. Findings from chloroplast data have further served as a suggestion that ‘*M. longifolia’* is the maternal parent of *M. spicata* [93,107,167].

However, our results show that *M. longifolia* is also polyphyletic and that accessions labeled as *M. spicata* (s.l.) consist of a composite taxon, both genetically (Figure 1) and morphologically (Figure 7A). Our genotype–phenotype analysis suggests a separate clade that can be interpreted as *M. spicata* s.s., while other samples formerly labeled *M. spicata* (s.l.) reside under either *M. longifolia* s.s. or *M. suaveolens* s.l. We find no evidence that *M. spicata* s.s. has any *M. suaveolens* ITS alleles, which is peculiar if the species indeed is a true alloploid involving the latter as one of the parents. Hence, the argument that concerted evolution makes *M. spicata* ITS sequences closely related to *M. suaveolens* [66] is not supported here. With respect to this, we stress that the taxon known as ‘Moroccan mint’, which has traditionally been erroneously perceived as *M. spicata,* is genetically and phenotypically clearly distinct from *M. spicata* (Figure 6). It is found within *M. suaveolens* s.l., which might have added to the concerted evolution hypothesis on ‘*M. spicata’*.

In conclusion, we do not find any evidence of a hybrid or cultivated origin of *M. spicata*. The subspecies ‘*glabrata’*, as defined by Lebeau [52,53,54] and associated with cultivation, is just another polyphyletic construct that is situated in our phylogeny as part of a genetic mix of glabrous to extremely hairy forms within *M. spicata* s.s. (cluster 2.5; Figure 1). Besides, in practice, glabrous variants are also part of totally wild populations. Last but not least, Drew et al. [123] put basal phylogenetic splits within sect. *Mentha* as occurring in the period from the late Miocene to the Quaternary, several million years ago. Therefore, it seems quite improbable the *M. spicata* s.s. complex or any of the species within would be mainly the result of post-Ice Age human in(ter)vention.

We do, however, find about 15% phenotypic overlap among our samples from both the *M. spicata* and *M. suaveolens–longifolia* complexes, and hence, it is possible that some gene flow might have occurred between the two complexes (certainly in the case of cultivars), which may have confused taxonomic assignments in the past. However, this gene flow might have occurred in more occasional, less systematic and multidirectional ways. Given our results, we strongly recommend that the taxonomic status of *M. spicata*, including its lectotype, should be revised. A more suitable candidate for the lectotype might be specimen S-LINN-IDC 238.1 denoted by ‘2 viridis’ in the Linnean herbarium (or alternatively LINN 730.5).

## 4. Materials and Methods

### 4.1. Sampling

We used mint accessions from ‘Bright Mints’, a permanent collection of living plants containing a large number (>500) of mainly European specimens of Spicate mints. That collection was largely built up by field sampling directed by known historical locations identified on a variety of herbarium vouchers from Paris (P, France), Meise (BR, Belgium), Leiden (L, the Netherlands), Kew (K, United Kingdom), Genève (G, Switzerland), Namur (NAM, Belgium) and Copenhagen (C, Denmark). Additionally, samples from other collections or from garden centers were included. We selected accessions to maximize the phenotypic and genotypic variability. The main target species were *M. spicata* (including *M. microphylla*), the hybrid complex *M. × rotundifolia–M. × villosa–M. × cordifolia* and *M. longifolia* (all sensu lato) from Europe. We also included some accessions from geographically distant areas (in particular accessions from NCGR, Corvallis, US; Table S1).

Specimens were selected to represent the largest possible phenotypic variation using a number of criteria: (1) assuring large geographical spread among selected specimen and taxa, (2) including historically described taxa, (3) ensuring large phenotypic range, (4) mostly excluding repeatedly hybridized or sterile cultivars to avoid potential bias caused by human selection and (5) including 10 voucher specimens (see below) for which ribosomal sequences were available. The final selection included 90 accessions (Table S1). Additionally, two herbarium specimens (one equivalent to the lectotype of *M. spicata*, BM00064600) were also included in the genetic analysis but were not morphologically assessed. A further 69 ribosomal sequences from voucher specimens were retrieved and included in the phylogenetic analyses (Table S1). An additional sample of *M. aquatica* L. was sequenced but not morphologically evaluated. In total, this resulted in 162 different specimens or vouchers and finally in 212 different sequences genotypically evaluated, which is, as far as we know, the largest (Spicate) mint dataset compiled to date.

To minimize environmental and plasticity effects on the morphological assessment, all the acquired plants were grown under the same common garden conditions (SE of Antwerp, Belgium; 51°03′ N, 4°46′ E). The plants were potted in 10 L pots containing potting soil (‘DCM universal’: dry matter 30%, organic matter 20%, NPK 1 kg/m^3^, pH 5–6.5), with the addition of loam soil (volume ca. 30%) and approximately 200 g chalk grains (‘DCM granular marine fossil coccolites’, 45% CaO, 2.5% MgO) to stabilize pH. The plants were grown under a clear sky for at least one full season (10–16 months), ensuring they had sufficient amounts of water. At the start of May, extra nitrogen–potassium solution was provided (liquid N/K 6:3, diluted 1:200). To prevent damage or abnormal growth, the collection was treated with pesticides (fungicide: ‘bio-cuprex garden’ containing 50% H_3_ClCu_2_O_3_; insecticide: ‘Edialux Bio-Pyretrex Garden’) and snails were systematically removed.

### 4.2. DNA Extraction and Sequencing

For 10 of the 90 samples in the living collection, ribosomal sequences were already available (Table S1). For the remaining 80 samples, fully extended leaves were selected and up to 100 mg of material was fast frozen in liquid nitrogen and powdered with a mortar under a sterile PCR hood to avoid sample cross-contaminations. DNA was extracted using the NucleoSpin Plant II kit (Machery-Nagel) following the manufacturer’s protocol. Preliminary experiments on a subset of samples showed difficulties with PCR amplification likely due to a large amount of PCR inhibitors, which is characteristic of aromatic plants. The DNA extracts were therefore diluted (20:1) prior to PCR amplification in order to limit the effects of such inhibitors.

Parts of the nuclear ribosomal region (ITS1-5.8S-ITS2) were PCR amplified using the forward primer 5′-AGAAGTCCACTGAACCTTATC-3′ and the reverse primer 5′-CGCTTCTCCAGACTACAATTC-3′ [168] in a 25 µL reaction volume containing 1X GoTaq reaction buffer (1.5 mM MgCl2), 0.2 mM of each dNTP, 0.5 µM of each primer, 0.5 U of GoTaq DNA polymerase (Promega), 10 ng of bovine serum albumin (BSA) and 2 µL (ca. 10 ng) of diluted genomic DNA. The amplification conditions were as follows: (1) initial denaturation at 94 °C for 2 min 30 s; (2) 40 cycles at 94 °C for 30 s, 54 °C for 30 s and 72 °C for 1 min 15 s; and (3) final extension at 72 °C for 10 min. The PCR products were visualized on a 2% agarose gel stained with Sybr Safe (ThermoFisher). The migration was performed for 50 min at 100 V and the picture was taken by a BioRad analyzer Gel Doc XR. PCR products were Sanger sequenced using the forward primer at Genewiz facilities (Leipzig, Germany). Samples with noisy chromatograms were resequenced using the reverse primer. The sequences were then assembled and phased using the software PHASE [169,170] and Champuru [147,171]. The analysis of the patterns of double peaks in the forward and reverse sequences allows haplotypes to be inferred without cloning.

Preliminary analysis using ITS sequences showed that some samples belonging to different taxa within *M. longifolia* s.s. shared sequences that could not be distinguished (see Section 2). To further characterize the genetic diversity and relatedness of these samples, we amplified the 10 nuclear start codon targeted polymorphism (SCoT) markers described by Khan and Dhawan [172] (see also [58,173]) from a subset of samples (Table S1). The amplification products were visualized on a 3% agarose gel, and the presence and absence of polymorphic bands were manually scored and summarized in a binary matrix.

### 4.3. Morphological Measurements

For all living specimens, a total of 122 morphological used variables (from which 63 variables are categorical and 59 are continuous; Table S2) were extracted from a set of 116 directly measured or scored features. Categorical variables were either binary (*n* = 11; mostly coded equivalents of multinomial variables) or ordinal (*n* = 52; concerning averages of twice independently scored values). In general, variables were measured on plants in full bloom (end of May/August and onwards depending on the species). However, for leaf shape measurements, leaves were sampled at least a few weeks before the plants started flowering and before mid-June. Twenty leaf-related variables were measured on a total of five to six scanned leaves (600 dpi scans fifth or sixth leaves from the top of plants) using Lamina 1.0.2 [174]. The mean of each continuous leaf variable was used for comparative analyses. Stems and flowers were simultaneously studied after selecting one well-developed, typical shoot/thyrse with cymes and flowers. One typical flower was chosen after microscopically (binocular 45×) evaluating the flowers of the whole plant. The flower characteristics of calyx and corolla were measured from photographs (CMEX5 DC.5000C) of dissected materials in ImageFocus 4.0.

Only above-ground parts of mints that concern characteristics of the calyx (*n* = 38) or other generative features (*n* = 17) were studied. Further, many leaf variables (*n* = 45) and some stem features (*n* = 8) were included. Other variables concerned general plant characteristics (*n* = 4) or organoleptic properties, i.e., odor (*n* = 10). Although some other features were also observed (e.g., stamen length, anther color and stigma development), they were not systematically assessed here because of their strong variability within the same taxon, which was apparent by inspecting the plants. We primarily preferred features with more inter- than intrataxon variability.

### 4.4. Data Analyses

#### 4.4.1. Genetic Analyses

Part of the ribosomal ITS region was sequenced for a total of 82 samples in this study (Table S1). In addition, 79 previously published ribosomal sequences from voucher specimens were retrieved from GenBank and included in the phylogenetic analyses (Table S1). All ITS sequences were aligned in MAFFT (E-INS-i parameters [175]), and a phylogenetic tree was reconstructed in BEAST v.1.10.4 [176] using 1 × 10^8^ chains and a GTR + I + G nucleotide substitution model, determined as the best-fit model (AIC) using jModelTest2 [177] with the following priors: κ: LogN [1, 1.25], initial = 2; frequencies: dirichlet [1,1]; clock rate: fixed, value = 1; tree model root height: tree prior in [0, Ꝏ], constant population size: 1/x, initial = 1. The burn-in was set to 1,000,000 and convergence was assessed in Tracer v1.7.1 [178]. The rooting of the tree was directed by the results, using a group of *M. arvensis−M. canadensis* samples as a posteriori outgroup (see Section 2). Phylogenetic clusters of sequences were identified using posterior probabilities (PP) > 0.6.

We further constructed a haplotype network using HaplowebMaker [179] to delimit fields for recombination (FFRs), i.e., species clusters [180,181,182]. We used all 90 living specimens, 2 herbarium specimens and a limited number of voucher specimens (Table S1). Based on visual inspection of the haploweb, genetic subclusters were identified.

The presence–absence data of SCoT amplification bands were converted to a matrix of Jaccard’s distances reflecting genetic dissimilarities across the samples. This and the distance matrix calculated from the ITS data were double factorized (principal components factoring, PCF; [183]), first separately and thereafter in combination (using original PCF-scores of the first two analyses). The resulting PCF biplot, therefore, shows the information contained in both the ITS alignments and the SCoT data, resulting in a further genotype distinction on the subcluster level.

Finally, genetic distances (K2P-GD) were calculated between the specimen units for samples included in the discriminant analyses (DA, see below). A crosstable is presented containing per unit combination the minimal observed GD value (GD_min_). Automatic barcode gap discovery (ABGD) for primary species delimitation [70] and GMYC [146] were used to estimate the range of the possible number of species, and the results were compared to those of the mentioned crosstable analysis.

#### 4.4.2. Morphological Analyses

Intercluster hybrid taxa, as identified by distinct sequences in different phylogenetic clusters, were excluded from the morphological analyses so as not to confound the analyses by human-induced selection or repetitively cross-bred strains. However, samples identified as *M. × rotundifolia* (L.) Huds. or hybrids with *M. × billotiana* (sensu Alfred Déségliseand Théophile Durand; hereafter as *M. billotiana*) were exceptions. Hybrids with *M. suaveolens* subsp. *insularis* (Req. ex Gren. & Godr.) Greuter or *M. suaveolens* subsp. *timija* (Coss. ex Briq.) were also included as these were the only representatives of a specific genetic cluster.

To evaluate the morphological space of the identified genetic subclusters, we choose a linear methodology and performed discriminant analysis (DA, R package ‘mass’ [184]) after having explored variable suitability by executing preparatory analyses such as penalized discriminant analysis on the full (122 variables) dataset as described below. Ward’s cluster method (*n* = 7, Euclidian distances; Table S2) was applied on a standardized variable subset after variable selection (variables with a correlation coefficient > 0.80 were removed to avoid multicollinearity effects; Table S2). The resulting Ward’s groups were projected in the DA space. Different relationships were assessed: the association (lambda) between Ward’s groups and specimen units (see below), the relationship (Kruskal–Wallis) between Ward’s groups and DA axes and the (visual) relationship between the Ward’s groups and existing plant labels.

For DA, we grouped plant samples genotypically as ‘specimen units’ based on recognized phylogenetic subclusters (PP > 0.6) and subclusters identified using the haploweb method and the SCoT analyses. DA maximizes the separation between predefined groups of samples (specimen units). Hence, morphotypes can be distinguished in a genotype-constrained space where genotypic specimen units are arranged according to their mutual morphological resemblances. Only lasso-selected independent variables entered the best model, and the magnitude and significance of Wilk’s lambda were tested (dispersion prerequisite: Δ_W_ < 0.05). Analyses were done in R 4.0.3. We evaluated the suitability of candidate variable subsets by different DA-performance-oriented criteria (proportion of between-unit dispersion (%) provided the lowest possible number of modeled variables (n_min_); representativity of DA-contained information for the whole dataset (Stewart–Love canonical redundancy index); and generic systematic (ranked) cohesion over the genotypic units (GLM-R^2^)) after selecting these candidate variable subsets out of the 122 variables by means of different lasso-penalized techniques (using packages ‘penalizedLDA’ [185] and ‘glmnet’ [186]) that outperformed ‘subselect’ [187] (results not presented here). The Stewart–Love canonical redundancy index, an index to measure the degree to which one set of variables can predict another set of variables, was calculated by relating the first six DA-dimensions based on the penalized variable subset with PCF (first six factors) including all 122 morphological variables (hence also binary variables [188,189]).

The idea behind this variable selection approach is to finally arrive, within a comparative DA-framework, at a limited subset of DA-modeled variables that perform well in the mentioned criteria without getting necessarily stacked up into one particular technique. Thereby, the lasso technique is especially aimed at reducing large variable sets (compared to sparse sample numbers) by penalization. When the lasso penalty tuning parameter (λ) is high, it leads to increased lasso penalties on, e.g., discriminant vectors, which generates sparse feature subsets in such penalized models. In DA, features with a strong within-class variability undergo greater penalization (creating a sparse classifier for which the decision rule involves only a subset of the features [190]). The minimum number of selected variables over all the penalized models puts the limit (n_min_) for included number of variables in the final DA.

Visualization was limited to the first three dimensions (gplot2 [191]). A 3D cube representation [192] is given to ease interpretation, more specifically in relation to the genetic main clusters.

Finally, a cluster analysis (centroid linkage) was done on the DA scores of the first three dimensions to pinpoint the different morphoclouds and, more specifically, those accessions that are morphologically overlapping while belonging to different genetic main clusters. The morphocloud analysis results were then plotted on the ITS phylogenetic tree to demonstrate correspondence and difference between the modeled morphology and ITS-SCoT genotype phylogeny.

## Figures and Tables

**Table 1:** Correspondence of Ward’s groups and traditional cryptic species perceptions using plant labels. Blocks coded by Ward’s groups are presented with genetic (sub)cluster codes and corresponding specimen units (plants). Ward’s groups can be compared to the available set of plant labels, accepting that for hybrid samples parent selection is done with the one parent most fitting the category becoming selected (‘label assignment’). Additionally, some explanation is given about the plant names as traditionally used. Color legend: see Figure 1.

Accession Number	Plant Labels (Only 1–2 Boxes Means Informal Label, 3 Boxes Means ‘Officially’ Labeled)			Label Assignment to One of the Parental Species (Criterium Least ‘Pattern Disturbing’ Parent)	Perceived Taxa	Morphotype (Ward’s Group Ranking)	Cluster ITS	Subcluster ITS + HAPL + SCOT	Specimen Unit
286					*M. spicata* (subsp. *glabrata*) or *M.* × *cordifolia*	1	4.2	4.2.2/4.2.1	4.2.2 × 4.2.1
455						1	2.5	2.5.2	2.5.2
562 (V)						1	2.5	2.5.2	2.5.2
627						1	1.0	-	1.0.0
217						1	2.5	2.5.1	2.5.1
555 (V)						1	4.2	4.2.1	4.2.1
499						1	2.1	-	2.1.0
222						1	4.1/4.2	4.1.1/4.2.1	4.1.1 × 4.2.1
409						1	4.1	4.1.1	4.1.1
570					*M. longifolia* incl. *M. asiatica, M. longifolia* subsp. *hymalaiensis*, *M. longifolia* subsp. *capensis*	1	2.3	2.3.2	2.3.2
53						2	4.1	4.1.1	4.1.1
564 (V)						2	2.2	-	2.2.0
567 (V)						2	2.2	-	2.2.0
571						2	2.3	2.3.1	2.3.1
595					*M. longifolia*	2	3.0/4.2	3.0/4.2.1	3.0.0 × 4.2.1
71						2	4.1	4.1.1	4.1.1
212						2	4.1	4.1.1	4.1.1
130						2	3.0	-	3.0.0
191						2	4.1	4.1.1	4.1.1
308						2	4.1	4.1.1	4.1.1
312						2	4.1	4.1.1	4.1.1
456						2	4.1	4.1.1	4.1.1
11						3	4.1	4.1.1	4.1.1
77					*M. longifolia* or *M. spicata* subsp. *tomentosa*/subsp. *condensata*/*M. microphylla*	3	2.5	2.5.1	2.5.1
250						3	2.5	2.5.2	2.5.2
283						3	2.5	2.5.1	2.5.1
319						3	2.4	-	2.4.0
311						3	4.1	4.1.1	4.1.1
248						3	2.5	2.5.1	2.5.1
306						3	4.1	4.1.1	4.1.1
450						3	4.1/4.2	4.1.4/4.2.3	4.1.4 × 4.2.3
313					*M. longifolia* (probably) or exceptionally *M. spicata* (?)	4	1.0	-	1.0.0
591						4	4.1	4.1.1	4.1.1
632						4	1.0	-	1.0.0
274					*M. longifolia* or hybrid	4	4.1	4.1.1	4.1.1
553						4	2.5	2.5.2	2.5.2
512						4	2.5	2.5.1	2.5.1
545						4	4.1	4.1.2	4.1.2
9						4	2.3	2.3.1	2.3.1
91						4	4.1/4.2	4.1.4/4.2.3	4.1.4 × 4.2.3
203						4	4.2	4.2.2	4.2.2
395						5	4.1/4.2	4.1.1/4.2.1	4.1.1 × 4.2.1
547						5	2.5	2.5.1	2.5.1
565 (V)						5	4.1	4.1.1	4.1.1
508					*M. spicata* subsp. *spicata* or *M.* × *villosa*	5	2.5	2.5.1	2.5.1
391						5	4.1/4.2	4.1.4/4.2.3	4.1.4 × 4.2.3
473						5	4.1/4.2	4.1.4/4.2.3	4.1.4 × 4.2.3
486						5	4.1/4.2	4.1.4/4.2.3	4.1.4 × 4.2.3
530						5	4.1	4.1.3	4.1.3
515						5	4.2	4.2.1	4.2.1
383						5	4.1/4.2	4.1.4/4.2.3	4.1.4 × 4.2.3
526						5	4.1/4.2	4.1.4/4.2.3	4.1.4 × 4.2.3
264					*M. suaveolens* (subsp. *suaveolens*) or its hybrids including *M.* × *villosa* (subsp. *alopecuroides*) and *M.* × *rotundifolia*	6	4.1	4.1.2	4.1.2
102						6	4.1/4.2	4.1.4/4.2.3	4.1.4 × 4.2.3
144						6	4.1/4.2	4.1.4/4.2.3	4.1.4 × 4.2.3
40						6	4.1	4.1.1	4.1.1
23						6	4.1	4.1.2	4.1.2
185						6	4.1/4.2	4.1.4/4.2.3	4.1.4 × 4.2.3
196						6	4.1/4.2	4.1.4/4.2.3	4.1.4 × 4.2.3
13						6	4.1	4.1.2	4.1.2
170						6	4.1	4.1.2	4.1.2
115					*M. suaveolens* subsp. *insularis* or *M. timija*	7	4.1/4.2	4.1.1/4.2.4	4.1.1 × 4.2.4
87						7	4.3/3.0	-	4.3.0 × 3.0.0
314						7	2.5	2.5.2	2.5.2
63						7	4.1	4.1.1	4.1.1
200					*M. suaveolens* (subsp. *suaveolens*) or *M.* × *rotundifolia*	7	4.1/4.2	4.1.4/4.2.3	4.1.4 × 4.2.3
190						7	4.2	4.2.2	4.2.2
76					*M. spicata* (subsp. *spicata*)	7	2.5	2.5.1	2.5.1
19						7	4.1	4.1.2	4.1.2
10						7	4.1	4.1.3	4.1.3

**Table 2:** Overview of genetic distances. Measured minimal values for genetic distances (GD_min_ as %) between specimen units. No intercluster hybrids are included. For unit 3.0.0, only nonhybrid samples were included. A mean value of average genetic distances between sister species was assessed by the method of Qin et al. [69] and estimated at 3.98% (95% confidence interval 3.36–4.59%). GD_min_ values are expected by us to be ca. 20% lower than the average.

Specimen Unit	1.0.0	2.1.0	2.2.0	2.3.1	2.3.2	2.4.0	2.5.1	2.5.2	3.0.0	4.1.1	4.1.4 × 4.2.3	4.2.1 ×	4.2.2	4.2.4 ×	4.3.0 ×
**1.0.0**	0.0														
**2.1.0**	14.9	0.0													
**2.2.0**	18.8	7.3	0.0												
**2.3.1**	12.9	3.4	2.9	0.0											
**2.3.2**	12.4	4.1	3.0	0.6	0.0										
**2.4.0**	12.7	4.7	3.2	1.2	1.5	0.0									
**2.5.1**	13.5	4.7	4.1	1.2	1.5	1.3	0.0								
**2.5.2**	14.3	5.0	4.4	1.5	1.8	1.6	1.0	0.0							
**3.0.0**	13.3	4.7	3.4	1.2	1.5	1.5	1.5	1.8	0.0						
**4.1.1**	12.8	4.7	3.0	1.2	1.5	1.5	1.5	1.8	0.	0.0					
**4.1.4 × 4.2.3**	13.3	5.3	4.1	1.8	2.1	2.1	1.9	2.4	0.6	0.7	0.0				
**4.2.1 ×**	13.1	5.0	3.9	1.5	1.8	1.8	1.8	2.1	0.6	1.0	0.1	0.0			
**4.2.2**	18.3	6.6	4.6	2.5	3.0	2.3	3.0	3.4	1.1	1.3	0.7	0.9	0.0		
**4.2.4 ×**	13.5	5.2	3.9	1.6	1.9	1.9	1.9	2.2	0.4	0.3	0.4	0.6	1.3	0.0	
**4.3.0 ×**	13.5	5.5	4.4	1.9	2.2	2.2	2.2	2.5	0.7	0.9	1.0	1.2	2.0	0.9	0.0
															
		interspecific													
		(in between)													
		sister species													
		(in between)													
		intraspecific													
		(no taxonomic consideration)													
															

## Data Availability

Sequences resulting from this study are deposited in GenBank. All other data not contained in the supplementary files are available upon request from the corresponding author.

## Supplementary Materials

The following material is available online at https://www.mdpi.com/article/10.3390/plants10040819/s1, Figure S1: Ward’s groups along the first three DA axes, Figure S2: Dendrogram of centroid linkage cluster analysis on DA scores, Figure S3: A generalized comparison of nrDNA phylogenetic results from different studies with an extract of the here presented phylogeny based on ITS. Figure S4: A generalized comparison of cpDNA phylogenetic results from different studies with an extract of the here presented phylogeny based on ITS. Table S1: Overview of accessions with their use and general information (GenBank, herbarium, etc.). Table S2: Overview of variables used for phenotype description.
