# Supplementary material for: A Revised Phylogeny of the Mentha spicata Clade Reveals Cryptic Species
Source: Plants (Basel). 2021 Apr 20;10(4):819. doi: 10.3390/plants10040819 (PMC8074783; doi:10.3390/plants10040819)
Supplement: Supplementary file 1 [file plants-10-00819-s001.zip › sup/Fig_S3.pdf]

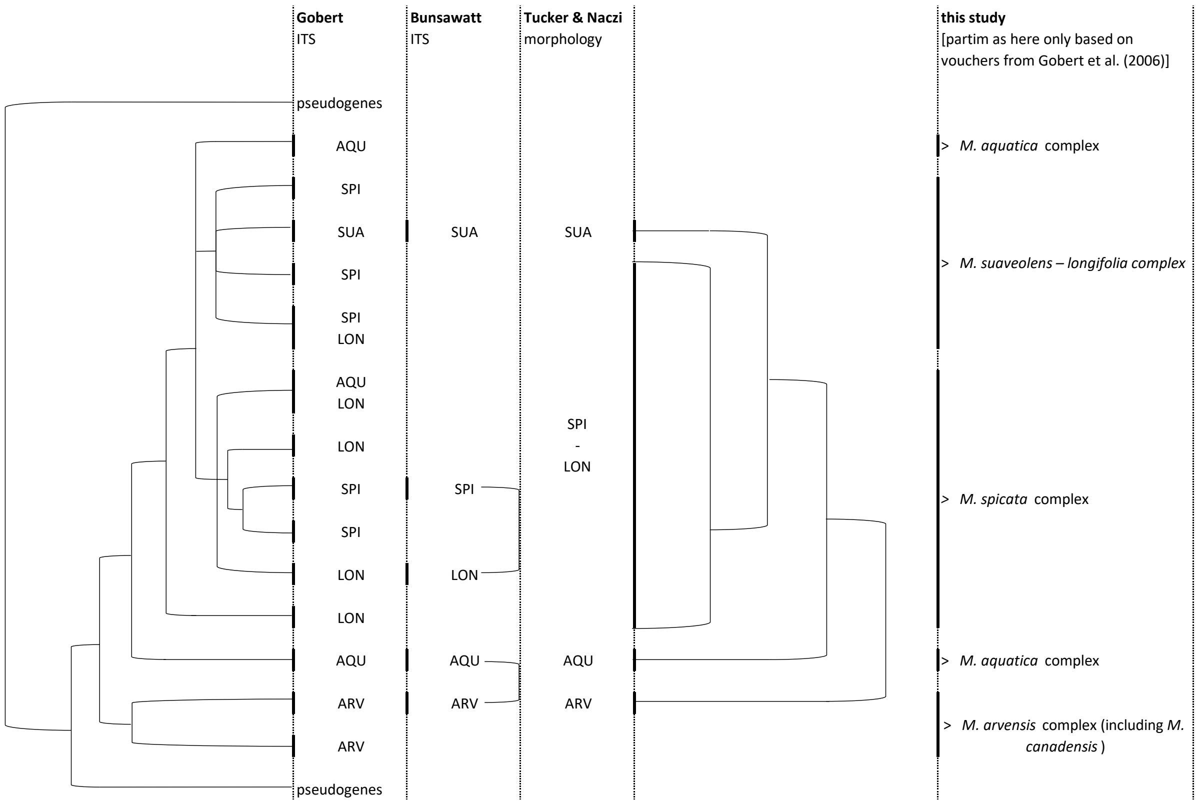

Legend :

AQU	*M. aquatica*
ARV	*M. arvensis*
LON	*M. longifolia*
SPI	*M. spicata*
SUA	*M. suaveolens*

(not represented: *M.* × *piperita* / *M.* × *canadensis*)
