# Supplementary material for: A Revised Phylogeny of the Mentha spicata Clade Reveals Cryptic Species
Source: Plants (Basel). 2021 Apr 20;10(4):819. doi: 10.3390/plants10040819 (PMC8074783; doi:10.3390/plants10040819)
Supplement: Supplementary file 1 [file plants-10-00819-s001.zip › sup/Table_S2.pdf]

Table S2 Overview of variables used for phenotype description

nr.	variable	type & unit	derived	selected variables (DA/Ward)
1	bract - length	C (mm)		W
2	bract hairs - density estimate	O (6 classes)		W
3	bracteole (lowest) - length	C (mm)		W
4	bracteole (lowest) - width	C (mm)		W
5	bracteole hairs - length	C (mm)		W
6	bracteoles - position (fully bending outward of spike = max.)	O (9 classes)		W
7	calyx hairs - density estimate	O (5 classes)		W
8	calyx hairs - length (max.)	C (µm)		
9	calyx hairs - length estimate of hairs at the lower side	O (4 classes)		W
10	calyx hairs - length estimate of hairs at the upper side	O (4 classes)		W
11	calyx hairs - max. length class (lower+upper)	O (4 classes)	from nr. 9-10	
12	calyx hairs - difference between upper and lower side length estimate	O (8 classes)	from nr. 9-10	W
13	calyx hairs - stiffness estimate	O (3classes)		W
14	calyx peduncle - length	C (mm)		W
15	calyx peduncle - width excluding hairs	C (µm)		W
16	calyx peduncle hairs - width of hairs over peduncle width	C (%)		W
17	calyx teeth 1-5 - length times width over area (average)	C (%)	originally from 20 measured variables: length, width, perimeter, area of calyx 1-5	W
18	calyx teeth 1-5 - length times width over area (trimmed average)	C (%)		
19	calyx teeth 1-5 - length times width over area (difference median and trimmed average)	C (%)		W
20	calyx teeth 1-5 - length times width over area (difference median and trimmed average over the trimmed average)	C (%)		
21	calyx teeth 1-5 - length times width over area (maximal difference 1-5)	C (%)		W
22	calyx teeth 1-5 - length times width over area (maximal difference 1-5 over the average)	C (%)		
23	calyx teeth 1-3 - length (average)	C (mm)		
24	calyx teeth 4-5 - length (average)	C (mm)		
25	calyx teeth 1-5 - length (maximum)	C (mm)		
26	calyx teeth 1-5 - length (minimum)	C (mm)		W
27	calyx teeth 1-5 - length (difference max.-min. over max.)	C (%)		DA / W
28	calyx teeth 1-3 - area (average)	C (mm^2)		
29	calyx teeth 4-5 - area (average)	C (mm^2)		
30	calyx teeth 1-5 - area (maximum)	C (mm^2)		W
31	calyx teeth 1-5 - area (minimum)	C (mm^2)		
32	calyx teeth 1-5 - area (difference max.-min. over max.)	C (%)		DA / W
33	calyx teeth 1-3 - perimeter (average)	C (mm)		
34	calyx teeth 4-5 - perimeter (average)	C (mm)		
35	calyx teeth 1-5 - perimeter (maximum)	C (mm)		
36	calyx teeth 1-5 - perimeter (minimum)	C (mm)		
37	calyx teeth 1-5 - perimeter (difference max.-min. over max.)	C (%)		
38	calyx teeth 1-5 - summed perimeters over summed areas	C (mm-1)		W

39	calyx teeth 1-5 - summed lengths over half of the summed perimeters	C (%)		W
40	calyx teeth 1-3 shape - triangle (perfect triangle = max., imploded triangle sides = min.)	O (6 classes)		
41	calyx teeth 4-5 shape - triangle (perfect triangle = max., imploded triangle sides = min.)	O (6 classes)		W
42	calyx teeth base 1-5 - corner at base of between-teeth interval (median)	C (degrees)		W
43	calyx teeth base 1-5 - inter-teeth shape (perfect U = max., perfect V = min.)	O (6 classes)		W
44	calyx tube - length	C (mm)		W
45	crown tube - length	C (mm)		W
46	crown colour - blue	O (RGB value)		DA / W
47	crown colour - green	O (RGB value)		
48	crown colour - red	O (RGB value)		W
49	crown colour continuity - from no distinct colouring, spots (small, medium, large), banding pattern (broken band, small band, strong band), to fully coloured	O (8 classes)		W
50	crown hairiness	O (3 classes)		
51	spike (main rachis) - conicity (fully conical = max., fully cylindrical = min.)	O (4 classes)		W
52	spike (main rachis) - contiguity (strongly interrupted = max., fully contiguous = min.)	O (9 classes)		W
53	spike (main rachis) - length	C (mm)		W
54	spike (main rachis) - width	C (mm)		W
55	spike cymes (main rachis) - number	C (n)		W
56	leaf appearance - leathery	O (3 classes)		W
57	leaf appearance - glabrosity (from almost glabrous to completely glabrous)	O (5 classes)		W
58	leaf asymmetry (average 5-6 leaves)	O (4 classes)		W
59	leaf colour - tinges of green (light to dark green) (average 5-6 leaves)	O (5 classes)		W
60	leaf colour (veil) - greyness (average 5-6 leaves)	O (5 classes)		W
61	leaf dimension - area (average 5-6 leaves)	C (mm^2)		
62	leaf dimension - center (horizontal) length (average 5-6 leaves)	C (mm)		W
63	leaf dimension - center (vertical) width (average 5-6 leaves)	C (mm)		W
64	leaf dimension - length (average 5-6 leaves)	C (mm)		W
65	leaf dimension - thickness (average 5-6 leaves)	C (mm)		W
66	leaf dimension - perimeter (average 5-6 leaves)	C (mm)		
67	leaf dimension - width (average 5-6 leaves)	C (mm)		W
68	leaf edge - sawtooth gentle (absent, weak, medium, strong)	O (3 classes)		W
69	leaf edge - sawtooth medium (absent, weak, medium, strong)	O (3 classes)		W
70	leaf edge - sawtooth rough (absent, medium, strong)	O (3 classes)		W
71	leaf edge - undulated-serrated (absent, medium, strong)	O (3 classes)		W
72	leaf hair - density (average 5-6 leaves)	O (8 classes)		W
73	leaf hair type - antler branched (absent, sparsely to frequent)	O (5 classes)		W
74	leaf hair type - multi-branched (absent, sparsely to frequent)	O (9 classes)		W
75	leaf hair type - single branched (absent, sparsely to frequent)	O (5 classes)		W
76	leaf hair type - softness (rigid over soft to velvet)	O (4 classes)		W

77	leaf hair type - stickyness (very sticky = max.)	O (5 classes)		W
78	leaf hair type - unbranched	binary		W
79	leaf hair type - woolliness (strongly woolly = max.)	O (5 classes)		W
80	leaf orientation - downward orientation	O (4 classes)		W
81	leaf shape - circularity (leaf area over squared perimeter in relation to unit circle) (average 5-6 leaves)	C (%)		W
82	leaf shape - curvature (strongly curved = max.)	O (9 classes)		W
83	leaf shape - guttershape	O (7 classes)		W
84	leaf shape - length over width quotient: minimal value of a) horizontal center over vertical center, b) horizontal center over vertical halfway (average 5-6 leaves)	C (%)		W
85	leaf shape - squared perimeter over area (average 5-6 leaves)	C (%)		W
86	leaf shape - logarithm of squared perimeter over area (average 5-6 leaves)	C (%)	from nr. 85	
87	leaf spots - redness	O (3 classes)		W
88	leaf teeth - bending backwards	O (3 classes)		W
89	leaf teeth - bending downwards	O (3 classes)		W
90	leaf teeth - bending sidewards (90°)	O (3 classes)		W
91	leaf teeth - depth (mean) (average 5-6 leaves)	C (mm)		W
92	leaf teeth - number per unit of leaf length (average 5-6 leaves)	C (mm-1)		W
93	leaf teeth - spacing (irregular)	O (3 classes)		W
94	leaf teeth type - gentle (absent, partly present, present)	O (3 classes)		W
95	leaf teeth type - medium (absent, partly present, present)	O (3 classes)		W
96	leaf teeth type - rough (absent, partly present, present)	O (3 classes)		W
97	leaf venation pattern - degree of anastomosis (from feather pattern with nerves of first degree to fully connected anastomosing network, waffled pattern = max.)	O (6 classes)		DA / W
98	petiole - height (average 5-6 leaves)	C (mm)		W
99	petiole - length (average 5-6 leaves)	C (mm)		DA / W
100	petiole - width (average 5-6 leaves)	C (mm)		W
101	internodium length - internod. 1 over internod. 2	C (%)		W
102	internodium length - internod. 2 over internod. 3	C (%)		W
103	stem - width (at internod. 6)	C (mm)		W
104	stem (base) colour - intensity (light to dark green)	O (3 classes)		W
105	stem (spot) colour - red (sparsely dotted to fully red)	O (4 classes)		W
106	stem (veil) colour - grey (weakly to strongly grey)	O (4 classes)		W
107	stem glands - number on section 2mm thick (at internod. 6)	C (n)		DA / W
108	stem xylem - ribs weak to strong	O (3 classes)		W
109	odour - appreciation	O (9 classes)		W
110	odour - strength	O (4 classes)		W
111	odour - artificial / weird	binary		W
112	odour type - carvon	binary		W
113	odour type - citrus	binary		W
114	odour type - menthol	binary		W
115	odour type - musty	binary		W
116	odour type - perfume incl. citrus	binary		W

117	odour type - stenchy	binary		W
118	odour type - thymol	binary		W
119	plant - habitus (strong, intermediary, weak) : strong	binary		W
120	plant - habitus (strong, intermediary, weak) : weak	binary		W
121	plant - height	C (cm)		W
122	plant - inflorescence length	C (mm)		
