# Supplementary figures and images for: A Revised Phylogeny of the Mentha spicata Clade Reveals Cryptic Species
Source: Plants (Basel). 2021 Apr 20;10(4):819. doi: 10.3390/plants10040819 (PMC8074783; doi:10.3390/plants10040819)

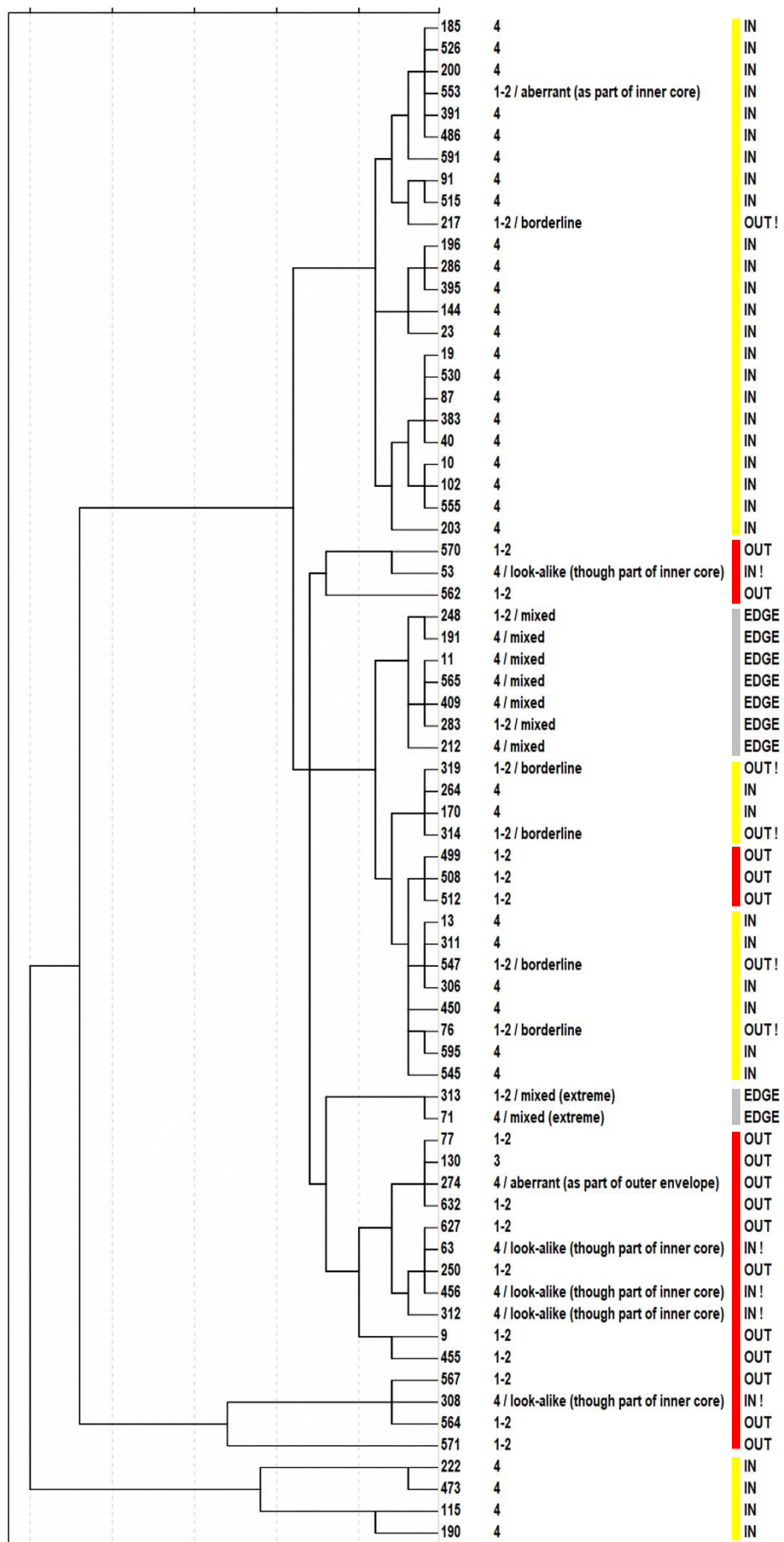

Supplement: Supplementary file 1 [file plants-10-00819-s001.zip › sup/Fig_S2.pdf]
